# Direct coupled electrical stimulation towards improved osteogenic differentiation of human mesenchymal stem/stromal cells: a comparative study of different protocols

**DOI:** 10.1038/s41598-024-55234-y

**Published:** 2024-03-05

**Authors:** João C. Silva, João Meneses, Fábio F. F. Garrudo, Sofia R. Fernandes, Nuno Alves, Frederico Castelo Ferreira, Paula Pascoal-Faria

**Affiliations:** 1 Department of Bioengineering and iBB-Institute of Bioengineering and Biosciences, Instituto Superior Técnico, Universidade de Lisboa, Av. Rovisco Pais, 1049-001 Lisboa, Portugal; 2 Associate Laboratory i4HB-Institute for Health and Bioeconomy, Instituto Superior Técnico, Universidade de Lisboa, Av. Rovisco Pais, 1049-001 Lisboa, Portugal; 3 Centre for Rapid and Sustainable Product Development (CDRSP), Polytechnic of Leiria, Marinha Grande, 2430-028 Leiria, Portugal; 4 Instituto de Biofísica e Engenharia Biomédica, Faculdade de Ciências, Universidade de Lisboa, Campo Grande, 1749-016 Lisboa, Portugal; 5 Instituto de Telecomunicações, Instituto Superior Técnico, Universidade de Lisboa, Avenida Rovisco Pais, 1049-001 Lisboa, Portugal; 6 Associate Laboratory for Advanced Production and Intelligent Systems (ARISE), 4050-313 Porto, Portugal; 7 Department of Mathematics, School of Technology and Management, Polytechnic of Leiria, Morro do Lena - Alto do Vieiro, Apartado 4163, 2411-901 Leiria, Portugal; 8 Department of Mechanical Engineering, School of Technology and Management, Polytechnic of Leiria, Morro do Lena-Alto do Vieiro, Apartado 4163, 2411-901 Leiria, Portugal

**Keywords:** Mesenchymal stem cells, Tissue engineering, Mesenchymal stem cells, Tissue engineering

## Abstract

Electrical stimulation (ES) has been described as a promising tool for bone tissue engineering, being known to promote vital cellular processes such as cell proliferation, migration, and differentiation. Despite the high variability of applied protocol parameters, direct coupled electric fields have been successfully applied to promote osteogenic and osteoinductive processes in vitro and in vivo. Our work aims to study the viability, proliferation, and osteogenic differentiation of human bone marrow-derived mesenchymal stem/stromal cells when subjected to five different ES protocols. The protocols were specifically selected to understand the biological effects of different parts of the generated waveform for typical direct-coupled stimuli. In vitro culture studies evidenced variations in cell responses with different electric field magnitudes (numerically predicted) and exposure protocols, mainly regarding tissue mineralization (calcium contents) and osteogenic marker gene expression while maintaining high cell viability and regular morphology. Overall, our results highlight the importance of numerical guided experiments to optimize ES parameters towards improved in vitro osteogenesis protocols.

## Introduction

Bone fractures are a global public health issue with a growing incidence among the elderly population. A systematic analysis conducted on worldwide available data (including 204 countries and territories) from 1990 to 2019 concluded that in 2019 there were 178 million new fractures (33.4% increase since 1990), 455 million prevalent cases of acute or chronic symptoms of a fracture (70.1% increase since 1990), and 25.8 million years lived with disability (65.3% increase since 1990)^1^. This data reflects a significant societal and economic burden predicted to be further aggravated by the worldwide population aging.

Bone tissue has the ability to regenerate upon fracture. However, defects larger than a critical size prevent the bone from self-healing and require further clinical intervention. Standard treatment includes sequential surgeries with autologous or allograft bone transplantation with the main disadvantages of donor site morbidity, and risk of immune rejection and pathogen transmission^2,3^. However, bone allografts are procured, processed, and distributed only by Tissue Banks operating under strict guidelines and sterile conditions, minimizing the risk of pathogen transmission. In order to surpass limitations from standard bone fracture therapy, bone tissue engineering (BTE) has emerged as a promising alternative strategy to regenerate functional bone tissue. BTE strategies often require the in vitro seeding of allogeneic or patient-derived (autologous) bone precursor cells into a scaffold structure that resembles and fits perfectly the bone defect shape. The cell-seeded scaffold can also be combined with chemical (e.g., growth factors) and physical (e.g., mechanical, electrical) stimulation to induce proper cell proliferation, osteogenic differentiation, and tissue maturation^4^. In a clinical translation context, the mature engineered bone construct would then be implanted into the patient to fill the fracture defect and boost local bone healing. Despite still being far from becoming a routine clinical strategy for bone repair, BTE protocols using cellular bone matrices (or cellular allografts) have been more frequently used in the surgical treatment of bone defects^5^. Such BTE strategies require a proper bone progenitor cell source that is easily accessible and can be expanded to clinically relevant numbers. Mesenchymal stem/stromal cells (MSCs) are a promising cell source for bone repair and have been playing a significant role in BTE strategies due to their ability to differentiate towards the osteogenic lineage, their high availability since they reside in many organs and tissues, their high in vitro proliferation capacity, low immunogenicity and advantageous immunomodulatory/trophic features^6,7^. Human bone marrow-derived MSCs (hBM-MSCs) are considered “gold standard” sources for cell-based therapies, and due to their nature as bone resident cells, hBM-MSCs have been widely used in BTE strategies^8–10^. Nonetheless, despite all the recent scientific advances and high potential of BTE strategies, a very limited number of products have reached the market of bone regeneration^11,12^, suggesting that current protocols still need to be improved and optimized.

Multiple electrical stimulation (ES) methods, including inductive, capacitive, or direct coupling, have been applied in BTE strategies to enhance cell proliferation and osteogenic differentiation^13^. Bioelectric cues function alongside chemical gradients, transcriptional networks, and haptic/tensile cues as part of the morphogenetic field that orchestrates individual cell responses^14,15^. ES has been used clinically for over four decades to promote bone healing, mainly as an adjunct to standard fracture care^16^. The application of a constant direct coupled (DCoupled) current stimulation protocol constitutes a simple, straightforward approach that has been shown to promote multiple cellular processes such as cell migration, proliferation, and osteogenic differentiation^17,18^. Several in vitro and in vivo studies have been conducted by applying DCoupled stimulation to undifferentiated stem cells and differentiated osteoblast cells through the use of different setups, electrode or substrate materials and a variety of waveforms^17,19,20^.

ES has been shown to activate multiple signaling pathways and molecules, such as bone morphogenetic proteins (BMPs), mitogen-activated protein kinases (MAPK), extracellular signal-regulated kinases (ERK), and p38. Also, voltage-gated calcium channels (VGCC) and integrins have been shown to play a significant role in transducing electric stimulation, leading to the upregulation of *Runx2*, the master regulator of the osteogenic differentiation pathway^17^. Accordingly, a study performed by Wang et al. demonstrated that a current-base DCoupled protocol (4 µA, 3 h per day for 14 days) enhanced significantly the proliferation and osteogenic differentiation of MC3T3-E1 preosteoblastic cells^21^. Moreover, Srirussamee and colleagues reported that a daily direct electrical stimulation of 2.2 V for 1 h during a total period of 7 days promoted the in vitro osteogenesis of human BMSCs, as evidenced by the significant upregulation of bone-specific marker gene SPP1^22^.

The mechanisms behind electrically-driven cellular processes rely on the modulation of membrane potentials by endo or exogenous electric fields (EFs)^20^. The scarcity of predictive models to guide and estimate the experimental EF delivered upon DCoupled stimulations has led researchers to rely on the applied input electric potential or electric potential drop between the electrodes as a stimulation magnitude comparator between different studies. However, these values do not translate the EF effectively delivered to cells since they do not take into account the critical dielectric properties of the materials involved, the geometry of the electrodes and stimulation chamber, or the complex electrode/electrolyte interface effects^23^. An exception was made by few studies modeling DC stimulation, Srirussamee et al.^22^ designed an electrochemical-based finite element method (FEM) model considering a secondary current distribution ruled by Ohm’s law that also accounts for charge transfer reactions, following the Butler-Volmer equation. Zimmermann et al. conducted two other studies exploring numerical modeling to optimize DCoupled ES protocols^24,25^. One tackled a DCoupled EF stimulation FEM model that solves Laplace’s equation and accounts for electrode-electrolyte interface interactions by adjusting the solution to experimental current measurements and by the previous calibration of model parameters with results from an electrochemical characterization^24^. Another work presents experimental and numerical methods to calculate the delivered EF and current density, including procedures with lumped-element and FEM model approaches^25^. Notably, the widespread implementation of digital models would be highly advantageous to improve current BTE methods since it will allow the optimization of stimulation protocols and develop predictive platforms while reducing experimental time and associated costs^26,27^.

The present study replicates a DCoupled setup originally conceived by Mobini et al.^28^ and further applied in subsequent studies^29–32^. Thus, distinctively from Mobini et al.^28^, we created a 6-well plate custom lid with L-shaped electrodes made of medical-grade stainless steel wire instead of pure platinum. Our work aimed to explore the influence of different ES parameters on MSC osteogenesis in vitro. Each applied protocol was guided by an electrical characterization of the DCoupled resultant waveform, in an attempt to understand the individual biological impact of the different parts of a typical stimulation waveform. The respective EF magnitude applied was predicted through finite-element modeling. These different protocols were then assessed in their ability to promote the osteogenic differentiation of hBM-MSCs. We explored applying a very short stimulation exposure (STIM2 OM) versus a more prolonged exposure (STIM1 OM), more typical of DCoupled stimulation studies. We also compared applying the same signal with two distinct frequencies (STIM4 OM and STIM5 OM) versus a steady electric potential step (STIM1 OM or STIM2 OM). Finally, a constant current-controlled condition (STIM3 OM) was explored and compared with all the other potential-controlled conditions by allowing one of the electrodes to float its potential accordingly. To the best of our knowledge, this is the first study to directly compare potential-driven ES with current-driven ES protocols to enhance the osteogenic differentiation of human MSCs towards improved BTE strategies.

## Results

### Electrode/electrolyte interface is mainly driven by faradaic processes

**Figure 1:**
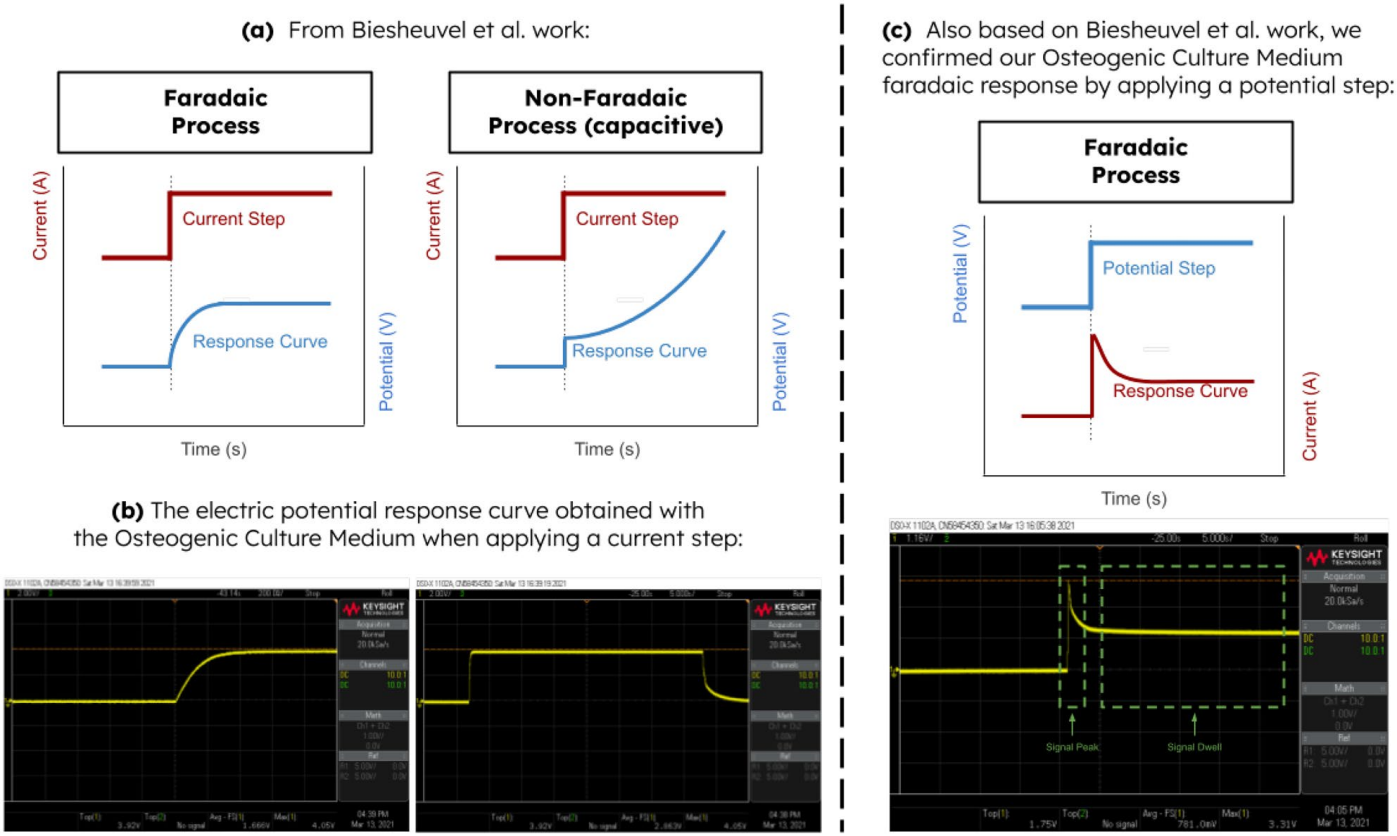
Response characterization of a DCoupled system accordingly to Biesheuvel et al.^33^ (**a**) Reference Faradaic versus Non-Faradaic expected responses for an applied current step; (**b**) Oscilloscope print screens for the measured responses of the custom developed DCoupled system for a current step input; (**c**) Reference versus oscilloscope print screen for the measured responses of the custom developed DCoupled system for a potential step input. In the potential step response (**c**), the peak and dwell phases of the waveform are identified. All oscilloscope print screens are made available at full-scale resolution in the supplementary materials.

**Figure 2:**
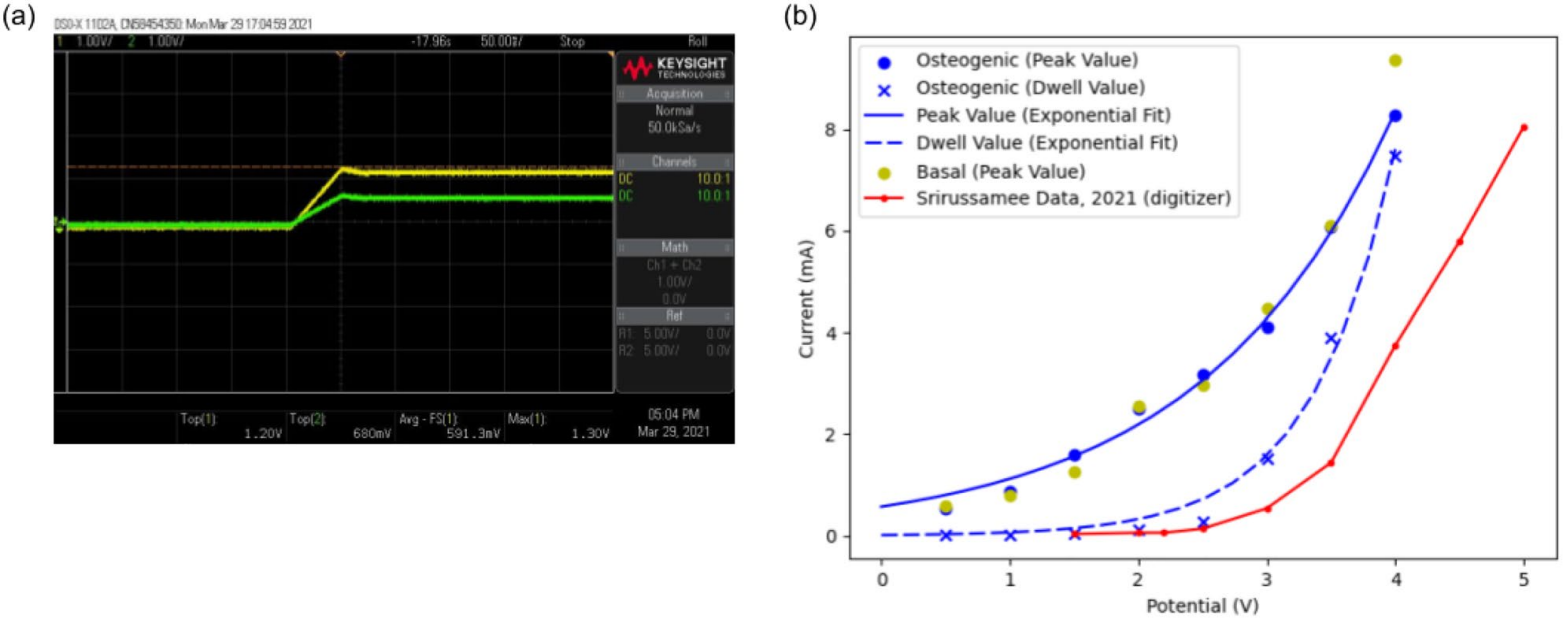
Response of the developed DCoupled setup with three wells in series: (**a**) Oscilloscope print screen for the potential step input response; (**b**) Comparison between the obtained I–V curve (maximum and stable forms, in blue) and curve data from Srirussamee et al.^22^ (in red; obtained with a digitizer).

The response of a DCoupled system single well (filled with osteogenic culture medium) to an electric current square wave input followed the predicted response for a faradaic process. Such behavior is distinct from a typical non-faradaic process as shown in Fig. 1a, b. Moreover, replacing the osteogenic culture medium with basal medium resulted in the same response curve shape, meaning that a faradaic process also drives basal medium interaction with stainless steel wire 316LVM. We further confirmed the electrode/electrolyte interface faradaic process by analyzing the response to an electric potential step since the response curve shape followed exactly the waveform reported by Biesheuvel et al.^33^ (Fig. 1c). This potential step response curve is characterized by an initial signal peak (higher potential and current) when the input potential step is applied, followed by a decrease in the electric potential until it reaches a stable signal dwell (lower potential and current). The developed system, composed of a series of three wells, showed the same response (Fig. 2a), just attenuated by the fact that the overall electric resistance of the system is at least three times higher than the electric resistance of a single well. The I–V curve for these three wells in a series setup is also shown in Fig. 2b. We also digitized the same curve obtained by Srirussamee et al.^22^ (red line in Fig. 2b) and observed that our stable curve and theirs are pretty similar in shape. The divergence observed in the values may be attributed to the different electrode/electrolyte species involved. We clarify that the stable curve in Fig. 2b represents the value of the electric current in the signal dwell phase. In contrast, the peak curve represents the maximum current achieved when the input signal is applied to the three wells in the series setup. The applied potential of 1.2 V was chosen deliberately below the water electrolysis limit of 1.23 V^23^ to test if DCoupled electric stimulation performed below the water electrolysis potential remains capable of producing similar osteoinductive effects as previously reported at higher potentials^29–31^. When 1.2 V is applied to our setup, we measured a peak electric current of 0.17 mA (during signal peak) preceding a current drop to 0.02–0.03 mA (during signal dwell). Dwell current showed to be slowly dropping with time. Regarding the electric current controlled hypothesis, a constant current condition of 0.03 mA generated electrode potential differences that were higher than the water electrolysis limit, registering a maximum potential difference of 3.74 V for one electrode pair in a three-well series. The results from this characterization process allowed us to create the protocol conditions described in the methods section, disaggregating the part of the waveform responsible for the higher current and higher EF from the part responsible for the lower current and lower EF.

### Computational modeling of the electric field within the ES setup

Before the FEM analysis of the electric fields generated within the ES setup, the electrical conductivities of both basal (BM) and osteogenic (OM) culture media were determined. For typical room temperatures of 21–23 °C, the electrical conductivity values were 1.383 S m^-1^ (OM) and 1.392 S m^-1^ (BM). Increasing the media temperature to 37 °C also increased its electrical conductivity to 1.741 S m^-1^ (OM) and 1.725 S m^-1^ (BM). The values measured follow the ones reported by Mazzoleni and colleagues^34^.

FEM solutions were obtained with conjugate gradients iterative solver that quickly converged (less than 10 s). Mesh independence was confirmed by repeating the calculation of the obtained results with the COMSOL extremely fine element size option. Slight geometrical deviations were introduced into the developed model to study the impact of such differences in the EF magnitude. Changing the distance between electrodes from 20 to 25 mm (while keeping the culture medium volume height constant and a constant electric current input) showed no difference in the electric field predictions larger than 0.01 V m^-1^, demonstrating that minor geometric deviations do not impact on this particular setup. The impact of culture media height changes was also studied (ranging from 2 mm to 6 mm in steps of 1 mm) at the same constant electric current magnitude and distance between the electrodes. The predicted electric field changes by almost 30% per milliliter of culture medium added or removed. This result indicates that each well should have the same volume of culture medium to equalize and assure the reproducibility of the stimulation conditions. In this study, we used 3 mL of culture medium per well of a standard 6-well culture plate, corresponding to a height of approximately 3.5 mm. At a constant electric current and culture medium height, changing the electrode length to more or less 5 mm will have a maximum impact of 6% in the predicted electric field. Also, under the same conditions, twisting 5^∘^ up/down one or both electrodes corresponds to a maximum variation of 0.09% in the predicted electric field, representing only a residual impact in this setup. More details on the numerical model methods and results are available in the supplementary materials.

**Figure 3:**
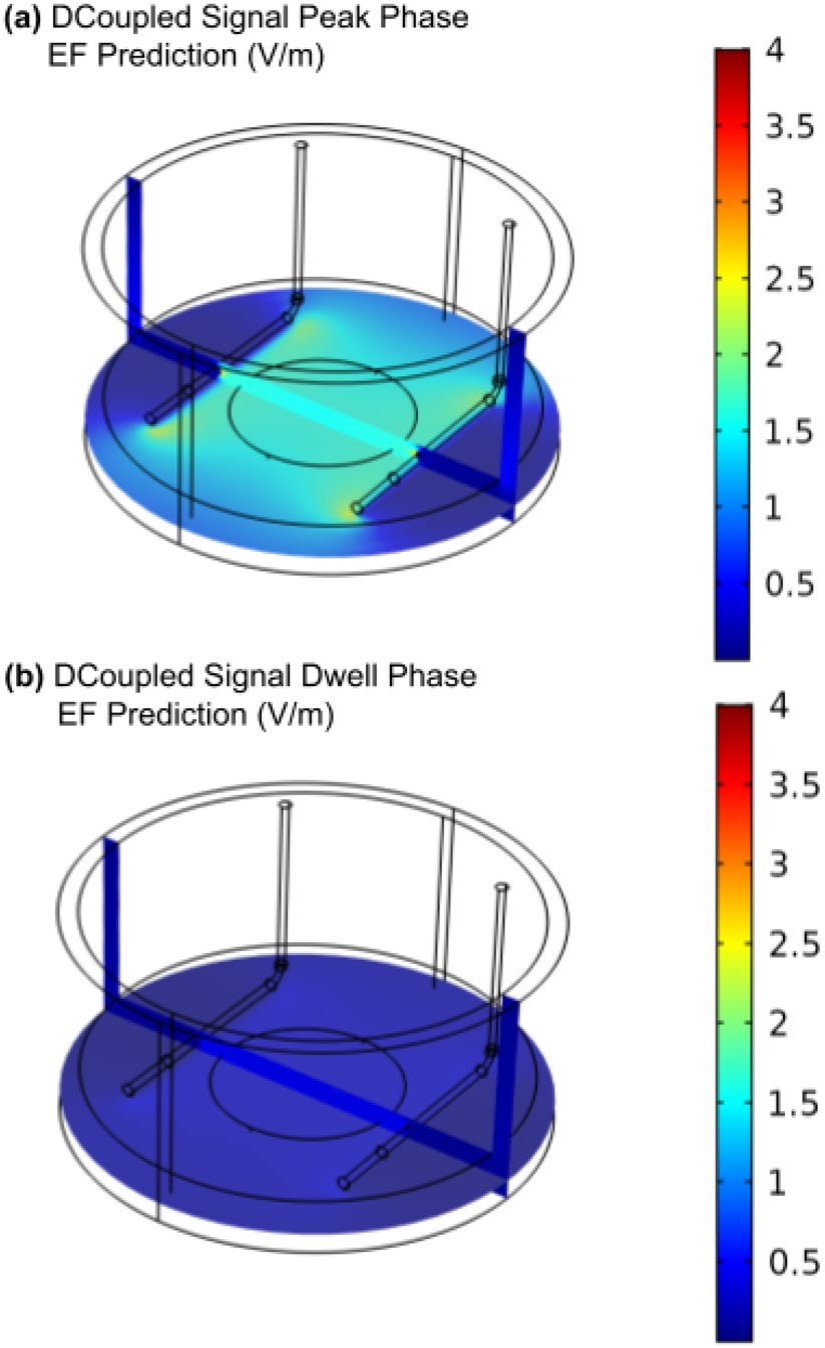
FEM numerical model of the developed DCoupled setup: (**a**) EF prediction for the signal peak electric current of 0.17 mA; (**b**) EF prediction for the signal dwell electric current of 0.03 mA.

Numerical FEM models of the developed setup predicted an average culture medium electric field in each of the three wells of 1.48 V $m^{-1}$ during the signal peak phase, and 0.26 V $m^{-1}$ during the signal dwell phase (Fig.3a, b).

### Effects of ES protocols on MSC viability, morphology and proliferation

**Figure 4:**
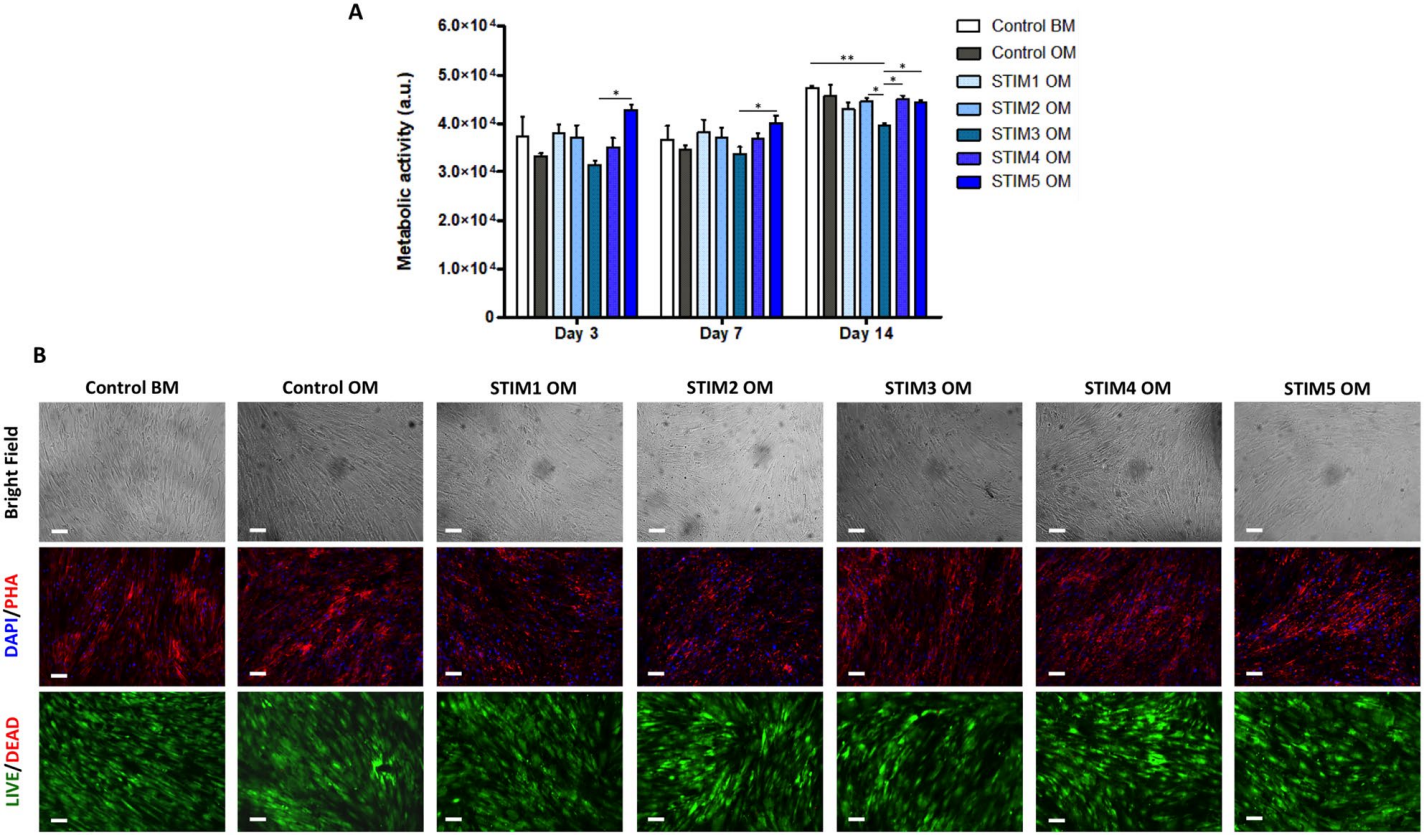
Effects of the different ES protocols on hBM-MSC’s metabolic activity, viability, and morphology. (**A**) Metabolic activities (determined using the AlamarBlue assay at days 3, 7, and 14) of hBM-MSCs undergoing osteogenic differentiation under the different ES protocols. Results are presented as average ± SD ($$n=3$$). $$*p< 0.05; **p < 0.01$$. (**B**) Assessment of cell morphology and viability by Bright Field imaging (top), DAPI/Phalloidin (middle), and LIVE/DEAD (bottom) fluorescent stainings at the end of the experiment (day 14). DAPI stains the cell nuclei blue, and Phallodin stains the actin cytoskeleton red. In the LIVE/DEAD staining, viable cells are stained in green, while dead cells appear in red. Scale bar: 100 µm.

The metabolic activity and viability of differentiating hBM-MSCs cultured under the different ES protocols for 14 days were evaluated using the AlamarBlue and LIVE/DEAD assays, respectively. As it is possible to observe in Fig. 4A, all the ES conditions promoted the maintenance of high metabolic activities throughout all the analyzed time points, similarly to the non-stimulated controls. However, statistical differences in cells’ metabolic activity were observed between the protocols, particularly on day 14 and concerning protocol STIM3 OM (current-driven condition). Additionally, all the ES protocols resulted in cultures with high cell viability (the presence of dead cells was residual) and regular morphology as demonstrated by LIVE/DEAD and DAPI/Phalloidin stainings, respectively (Fig. 4B).

### ALP activity, calcium production, and osteogenic stainings

**Figure 5:**
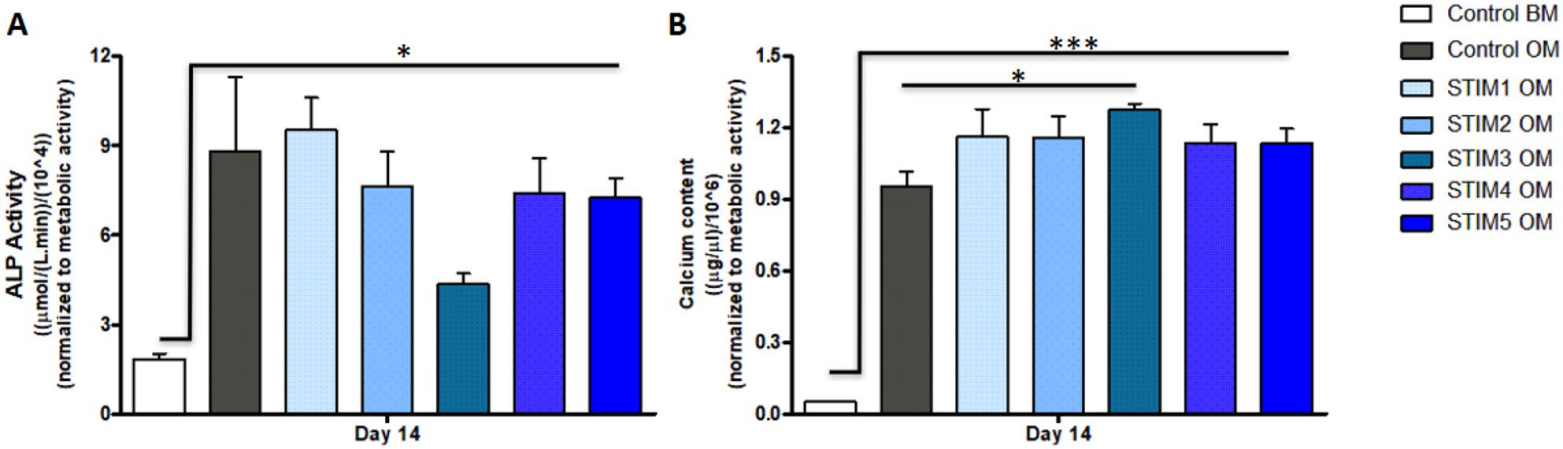
(**A**) ALP activity and (**B**) calcium deposition quantification of hBM-MSCs cultured under five different ES protocols after 14 days of osteogenic differentiation. Results are presented as average ± SD ($$n=3$$). $$*p< 0.05; ***p < 0.001$$.

To assess the effects of the different ES protocols on the osteogenic differentiation of hBM-MSCs, Alkaline phosphatase (ALP) activity (Fig. 5A) and calcium content (Fig. 5B) quantitative assays were performed on the cultures obtained at the end of the experiment (day 14). As expected, all the conditions (ES and non-stimulated) cultured under osteogenic medium presented statistically significantly higher ALP activities than the non-stimulated cells cultured under standard growth media conditions (CONTROL BM). Lower ALP activity values were observed for the current-based protocol (STIM 3 OM). Nevertheless, this difference was not statistically significant (Fig. 5A).

Regarding mineralization, the experimental groups cultured under osteogenic induction conditions present significantly higher calcium contents than the CONTROL BM group (Fig. 5B). Notably, the current-based ES protocol (STIM 3 OM) was the only ES condition that achieved statistically significant higher calcium content (mineralization) than the non-stimulated cells cultured under osteogenic medium (CONTROL OM).

**Figure 6:**
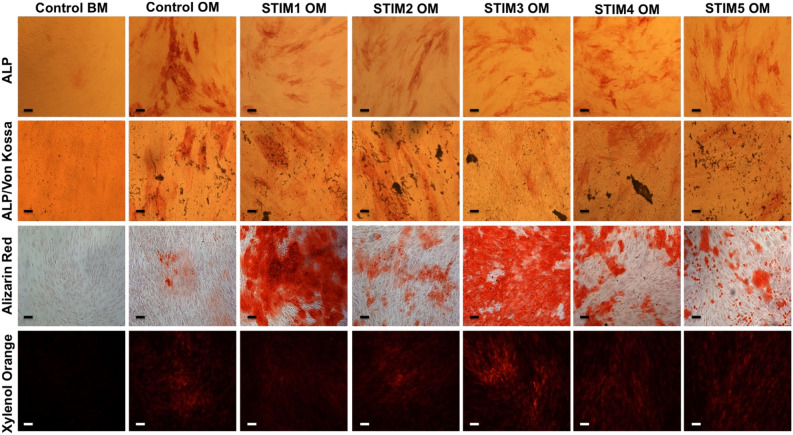
ALP, ALP/Von Kossa, Alizarin Red, and Xylenol Orange stainings (from top to bottom rows) of hBM-MSCs cultured under osteogenic differentiation conditions and exposed to the different ES protocols for 14 days (and respective non-stimulated controls). ALP/Von Kossa staining evidenced the ALP activity of the differentiating hBM-MSCs (reddish areas) and mineralization (Von Kossa–dark deposits). Alizarin Red staining further confirmed the presence of calcium deposits (red staining). Xylenol Orange fluorescent staining showed the presence of calcium deposits in red. Scale bar: 100 µm.

The differentiation of hBM-MSCs towards osteoblasts was further confirmed by ALP/Von Kossa, Alizarin Red, and Xylenol Orange osteogenic stainings (Fig. 6). As expected, all the experimental groups cultured under osteogenic induction medium stained positively for ALP activity (top row) and cell mineralization (three bottom rows), demonstrated by the presence of black, red, and fluorescent red mineralized deposits identified by Von Kossa, Alizarin Red and Xylenol Orange stainings, respectively. None or minimal positive osteogenic stainings were observed for the CONTROL BM group. Importantly, concerning the Alizarin Red staining, all the ES protocols showed a more intense and spread staining than the osteogenic non-stimulated group (CONTROL OM). This was particularly evident for the STIM1 OM and STIM3 OM experimental groups. Moreover, Xylenol Orange staining images also suggest a more intense staining for the current-based ES protocol (STIM3 OM).

### Osteogenic gene expression and immunofluorescence analysis of bone-specific proteins

**Figure 7:**
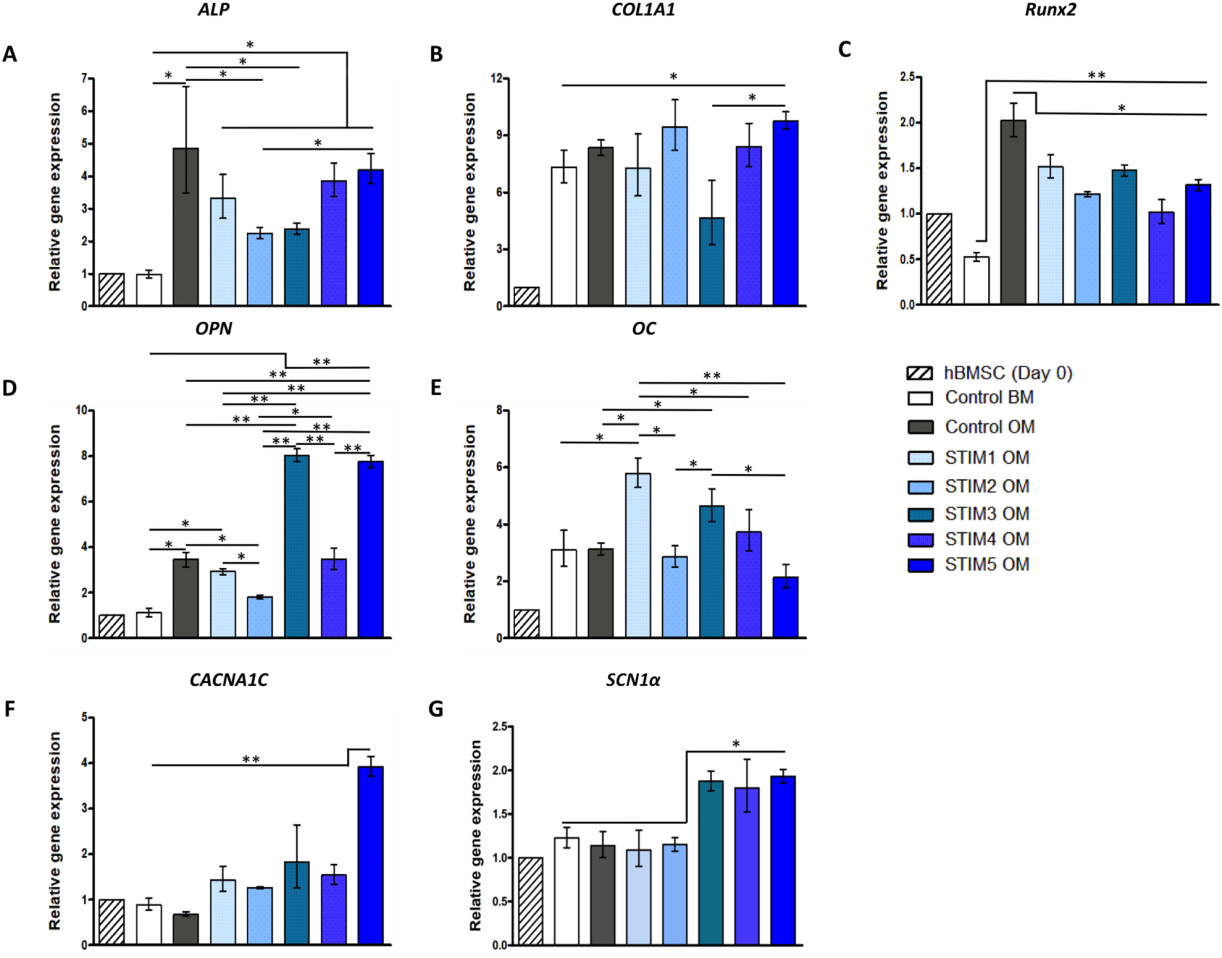
Gene expression analysis by RT-qPCR of hBM-MSCs undergoing osteogenic differentiation under the different ES protocols for 14 days. Expressions of (**A**) *ALP*, (**B**) *COL1A1*, (**C**) *Runx2*, (**D**) *OPN*, (**E**) *OC*, (**F**) *CACNA1C* and (**G**) *SCN1*$$\alpha$$ were normalized to two reference genes (*GAPDH* and *RPL13A*) and calculated as a fold-change relative to the baseline expression of the control sample (hBM-MSCs at day 0). Results are expressed as average ± SD of three ($$n=3$$) independent samples. $$*p< 0.05; **p< 0.01$$.

RT-qPCR analysis was performed to evaluate the effects of the five different ES protocols on the expression of bone-specific genes by hBM-MSCs cultured under osteogenic induction medium for 14 days. As it is possible to observe in Fig. 7, marker gene expressions were distinctively influenced by the different ES protocols. *ALP* (Fig. 7A) expression was the highest for the non-stimulated CONTROL OM condition. Still, this difference was only statistically significant concerning STIM2 OM and STIM3 OM groups. All the experimental groups cultured in an osteogenic medium presented significantly upregulated ALP expression in comparison to CONTROL BM. *COL1A1* (Fig. 7B) expression was significantly higher in the STIM5 OM condition only in comparison to CONTROL BM and STIM3 OM. Moreover, statistically significant differences in *COL1A1* expression were not observed between all the other protocols. As expected, the expression of *Runx2* (Fig. 7C) was significantly higher in all the groups cultured under osteogenic induction in comparison to the basal medium condition (CONTROL BM). However, the CONTROL OM group achieved a significantly higher *Runx2* expression than all the ES protocols. *OPN* (Fig. 7D) expression was upregulated in all samples cultured in osteogenic medium in relation to the control BM group, with the exception of STIM2 OM protocol. Notably, the expression of this late-stage differentiation marker (*OPN*) was significantly higher in the protocols STIM3 OM (current-driven) and STIM5 OM when compared to all the other experimental groups cultured under osteogenic induction conditions. *OC* (Fig. 7E) expression was the highest in cells cultured under the STIM1 OM protocol. Such upregulated expression was statistically significant compared to all experimental groups, except for the STIM3 OM protocol. The gene expressions of *CACNA1C* and *SCN1*$\alpha$ - which encode for the 1C subunits of type L of voltage-gated calcium channels and 1$\alpha$ subunit of voltage-gated sodium channels, respectively—were also analyzed due to their known role on the delivery of electrical cues to cells^35^. *CACNA1C* (Fig. 7F) expression was significantly upregulated in the STIM5 OM condition presenting a significantly higher expression levels than all the other experimental groups. Intriguingly, *SCN1*$\alpha$ (Fig. 7G) expression was only significantly upregulated (comparatively to the non-stimulated groups) in the protocols STIM3 OM, STIM4 OM, and STIM5 OM.

**Figure 8:**
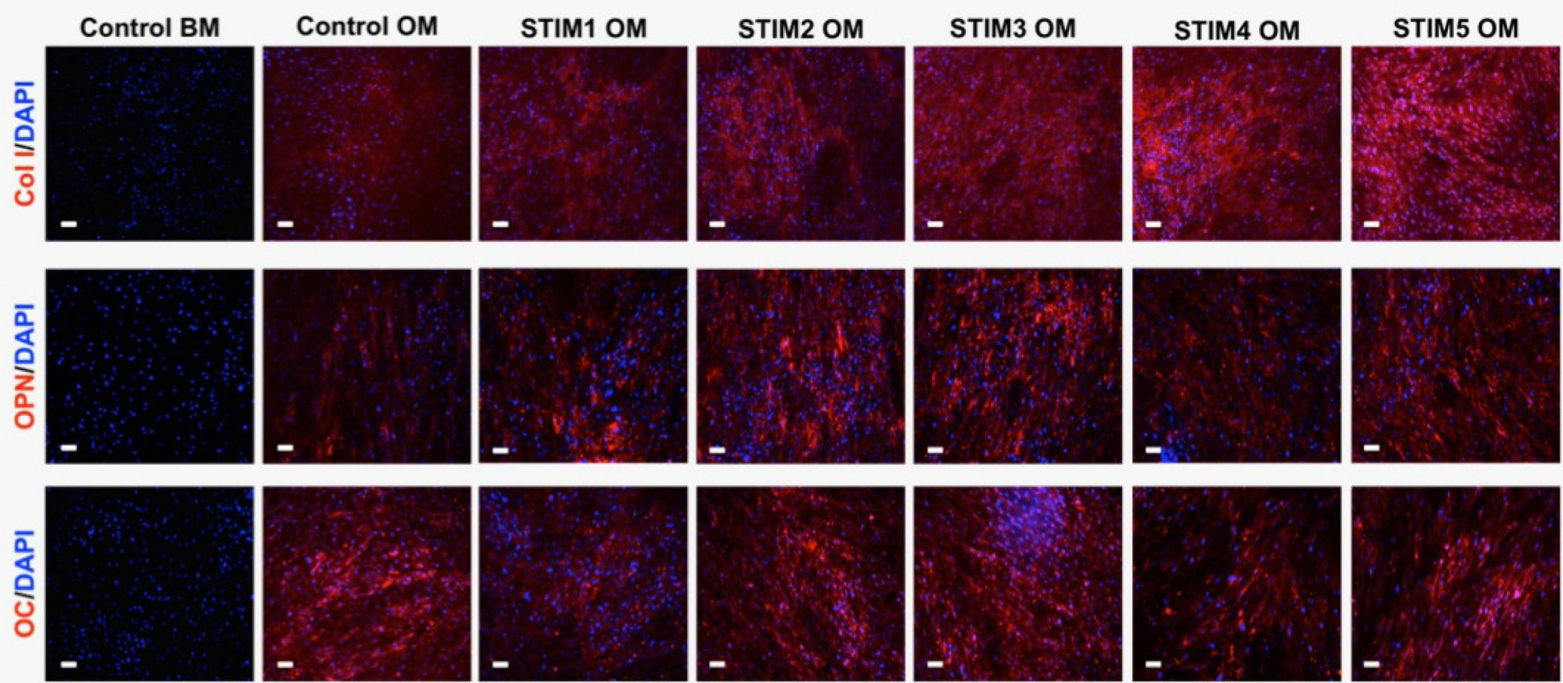
Immunofluorescence analysis to evaluate the presence of type I collagen (Col I, top), osteopontin (OPN, middle), and osteocalcin (OC, bottom) bone-specific proteins on differentiating hBM-MSCs cultured under osteogenic induction conditions and exposed to the different ES protocols for 14 days (and respective non-stimulated controls). Antibody fluorescent staining in the samples appears in red. The samples were counterstained with DAPI, which stains cell nuclei in blue. Scale bar: 100 µm.

Immunofluorescence staining of the samples obtained after the osteogenic differentiation of hBM-MSCs exposed to the five different ES protocols was performed to assess the presence of the relevant bone ECM proteins type I collagen, osteopontin, and osteocalcin. As shown in Fig. 8, all three proteins (Col I, OPN, OC) were positively identified in all the experimental groups cultured under osteogenic induction conductions, with no noticeable differences in marker intensity expression being observed between the different ES protocols.

## Discussion

An essential difference between direct-coupled systems that apply their EF through electrode/electrolyte interfaces (the system used in this work) and those that apply the EF through agar salt bridges^18^ is that in the latter, only potassium and chloride ions flow from the agar salt bridge into the cell culture region. By contrast, when using immersed electrodes, uncontrolled and unknown free-flowing ions balance the charges by becoming oxidized and reduced in the process, which can unleash unexpected biological effects^22,36^. The electrode may release ions from its material, generating free radicals or being biologically active without further reactions. Stainless steel electrodes have been previously reported to release ferrous ions when subjected to pulsed stimulation. The ferrous ions release was strongly correlated with waveform parameters and the medium’s ionic strength^37^. Since stainless steel electrodes can not be considered inert, small constant electric currents or potential waveforms are expected to release small ionic content into the media, an effect neglected in this current study. Our study unveils the predominant faradaic nature of the electrode/electrolyte interface between the stainless steel 316LVM and the medium (BM or OM), which was established by applying a simple characterization process described by Biesheuvel and coworkers^33^. Since our observations result from applying electric potentials inferior to the water hydrolysis limit, we can infer that other cell culture medium chemical species are being oxidized/reduced. This reinforces the importance of further understanding the faradic by-products that are being produced in DCoupled stimulations when the electrode material is in direct contact with the culture medium, a necessity also highlighted by Srirussamee et al.^36^ and Tomov et al.^37^. The average electric fields generated by our DCoupled setup are predicted to be between 1.48 and 0.26 V m^-1^, corresponding to the time-dependent system response to the input potential step signal, generating a peak electric current (0.17 mA) that immediately drops to a much lower current (0.02–0.03 mA). This peak effect is only observable when applying an electric potential step, since when applying an electric current step, the current source will adjust the electric potential of the electrode terminals in a time-dependent manner. Once the system becomes more/less resistive, the current source will decrease/increase the electric potential at the electrode terminals to maintain the established current. The original DCoupled setup from Mobini et al.^28^ was subjected to a current measurement validation in a later work by Srirussamee and colleagues^36^. When the authors applied Mobini’s potential of 2.2 V DC, a total current of 0.07 ± 0.01 mA (mean ± SD) was measured in each well after it reached a steady state. This measurement corresponds to the dwell phase of the system signal response, as we have observed here. Despite being of identical magnitude to our measured current at the same signal phase (0.02–0.03 mA), the observed difference may arise from the differences in the electrical conductivity of the used culture medium and on the electrode material, which in turn generates a different electrode/electrolyte I–V curve interface relation, as shown in Fig. 2.

Since our developed DCoupled setup tries to emulate Mobini’s DCoupled setup, we also numerically modeled Mobini et al. setup^28^ using the same methodology, considering their reported geometry and the posterior measured electric current by Srirussamee et al.^36^. We also took advantage of known typical electrical material properties for platinum, polystyrene, and culture medium (described in the supplementary materials). For those boundary and material conditions, the developed model predicts an average electric field for the Mobini setup of 0.59 V m^-1^ in the culture medium volume. This predicted electric field is lower than the reported by Mobini et al.^28^ original setup and by other subsequent studies using the same setup^29–31^, which report that a potential step of 2.2 V DC generated 100 V m^-1^. This value is contradicted by Srirussamee et al.^36^ current measures, later repeated with more detail^22^ and with an additional potential measure at two distant points (8 mm apart) in the culture medium.

From Srirussamee et al.^36^ Figure 2 and with the help of a digitizer, the voltage drop between the two points can be estimated to be 0.0169 V (A: 0.8125 V, B: 0.7956 V). This potential measurement allows us to grossly estimate the electric field to be 2.1 V m^-1^, a magnitude value closer to our estimate. The difference between this value and the ones presented in our study may rely on the unknowns of the exact properties of the culture medium and the exact geometry of the electrodes and liquid volume used, as we expose in supplementary materials. Nevertheless, the measurements from^36,37^ reinforce the confidence in our delivered EF prediction methodology. Also, the study from Zimmermann et al.^24^, describing the application of digital models to monitor and control electrical stimulation in vitro, considered a stimulation chamber similar to the Mobini et al.^28^ setup. Results from their DCoupled model for Mobini’s DC stimulation conditions predicted an EF of 0.33 V m^-1^, being followed by Srirussamee et al.^22^ current density predictions (0.5 A $m^{2}$) and by our model calculations for Mobini’s EF magnitude (0.59 V m^-1^), which is considerably different from the values of 100 V m^-1^ reported by Mobini et al. at their original setup and protocol. Regarding DCoupled stimulation regimes using directly immersed electrodes, agar-salt bridges, or a conductive scaffold substrate, a mandatory reading for further understanding of the electrochemistry effects in each setup is the theoretical analysis of Guette-Marquet et al.^23^. Their analysis also suggests that, for two-electrode systems (like the one used here), many reported EF magnitude values have been overestimated, which is also in line with Zimmermann et al.^24^ and with our predictions. Guette-Marquet et al. also advises changing experimental practices by applying current instead of voltage, a condition that we implemented in the protocol condition STIM3 OM. We controlled and reported the resultant electric current for the remaining potential conditions. Although we have used a direct probing method (multimeter) to measure the electric current that passes through the system, indirect probing methods should be privileged in the future to avoid direct influence in effects, like the Rogowski-coil method of measuring electric current^38^.

The successful clinical outcomes of ES in bone healing strategies encouraged the scientific community to try to understand its underlying mechanisms at both the cellular and molecular levels. ES has been previously applied to enhance the osteogenic differentiation of MSCs in vitro^17,39,40^. However, the cellular processes/signaling pathways by which ES regulates osteogenesis are still poorly understood. Therefore, there is no optimal, defined, and standardized ES protocol for inducing MSCs osteogenic commitment in vitro. Many studies using various poorly characterized experimental systems for ES limit the comparison of results and protocol reproducibility. Existing studies often use single voltage-controlled protocols to treat MSCs without using numerical modeling to optimize stimulation parameters. Results are often analyzed only by comparing them to non-stimulated controls, which difficult comparisons with other works. Moreover, applying current-based ES protocols imposing current intensity instead of voltage in two-electrode systems to study cellular processes is vastly unexplored^23^.

Thus, in this study, we aimed to directly compare different potential-controlled ES protocols with a current-controlled one in terms of their capacity to improve the in vitro osteogenesis of hBM-MSCs. The EF magnitude calculation performed will allow us to compare this work output with similar future studies. All the ES protocols resulted in final cell cultures with high viability, high metabolic activity and regular cell morphology (Fig. 4). These results follow the study from Zhao et al.^41^, which reported high cell viabilities (90–95%) and typical elongated fibroblast-like morphology for human BM-MSCs exposed to EFs of 200 mV mm^-1^ (two orders of magnitude higher than the EFs applied in this work). Regarding the applied current-controlled protocol (STIM3 OM), the overtime increase in metabolic activity (indirect measure of cell proliferation, AlamarBlue assay) observed in Fig. 4A and high cell viability/regular cell morphology (Fig. 4B) are concordant with the results reported by Shao et al.^42^ for osteoblasts exposed to DCoupled ES of 100 µA (4 h per day) for 6 days.

The effects of the different ES protocols on the hBM-MSCs osteogenic differentiation were assessed after 14 days through the quantification of ALP activity and calcium production (Fig. 5), typical osteogenic stainings (Fig. 6), bone-related marker genes expression (Fig. 7) and immunofluorescence analysis of essential bone ECM proteins (Fig. 8). Our results suggested an advantageous performance of the applied current protocol (STIM3 OM) in enhancing calcium production/mineralization by osteogenic differentiating hBM-MSCs (Fig. 5B). This result concurs with the more intense and spread Alizarin Red and Xylenol Orange stainings observed in Fig. 6 for the cultures exposed to STIM3 OM protocol. Moreover, the higher mineralization observed for the applied current ES condition is supported by the previous study performed by Zhang et al.^43^, in which significantly higher calcium deposition was obtained (at day 14) for human adipose-derived MSCs cultured on polypyrrole/polycaprolactone scaffolds under osteogenic induction and exposed to 200 µA of direct current for 4 h per day.

All the ES protocols resulted in similar or lower ALP activities (more pronounced in the STIM3 OM protocol, Fig. 5A) and *ALP* (Fig. 7A), *Runx2* (Fig. 7C) gene expressions than the non-stimulated Control OM group. Such observation might be explained by the fact that *Runx2* and *ALP* expressions (and respective ALP activity) are more predominant in the initial phase of MSC’s osteogenic differentiation (early markers) that precede the mineralization phase, after which their levels decrease^44^. Thus, it is possible that ES protocols (particularly STIM3 OM) promoted a faster MSC’s osteogenic differentiation process (as supported by the enhanced mineralization (calcium content in Fig. 5B and Alizarin Red staining, Fig. 6), upregulation of late-stage markers (*OPN* (Fig. 7D) and *OC* (Fig. 7E)) expression and respective proteins presence in Fig. 8), resulting in the observed lower ALP activity and *Runx2* and *ALP* gene expressions. Accordingly, our results are also in line with previous BTE studies reporting decreased ALP activity as a result of a more advanced osteogenic differentiation stage of the MSCs^45,46^.

OPN has been shown to play a pivotal role in regulating calcium phosphate nucleation during the mineralization process^47^. Thus, the significantly higher *OPN* gene expression observed in Fig. 7D for the cultures exposed to STIM3 OM protocol is well correlated with the enhanced mineralization obtained for the same condition (Figs. 5B and 6—Alizarin Red and Xylenol Orange stainings). This beneficial effect of applied current ES on *OPN* expression and mineralization has been previously reported^43^. *OC* gene upregulated expression has also been associated with improved bone mineralization^48^. Therefore, the significantly higher *OC* expressions obtained for the experimental groups STIM1 OM and STIM3 OM might explain these conditions’ improved calcium production (Fig. 5B) and more intense mineral deposition (Alizarin Red staining, Fig. 6).

*CACNA1C* gene role in the signaling cascade regulating the osteogenic differentiation of human MSCs and subsequent tissue mineralization has been demonstrated in previous works^43^. Zhang et al. showed the superior role of voltage-gated calcium channels in the modulation of adipose-derived MSCs in comparison to other ionic channels (sodium, potassium, and chloride)^43^. Moreover, the study from Camarero-Espinoza and Moroni further evidenced the correlation between *CACNA1C* and the osteogenic differentiation of hBM-MSCs, as they showed that blocking the activity of *CACNA1C* resulted in a downregulation of the bone-specific genes (*Runx2*, *COL1A1* and *OC*)^49^. Previous studies have proposed that ES promoted the increase of cytosolic calcium ionic concentration both in osteoblasts and MSCs, which subsequently activates voltage-gated calcium channels and regulates cell functions via calmodulin pathways^43,50^. Accordingly, our results showed that all the ES protocols resulted in the upregulation of *CACNA1C* expression (Fig. 7F) with the highest levels observed for the STIM5 OM condition. STIM5 OM protocol also promoted the highest *COL1A1* expression (Fig. 7B), which is in line with the relation between *CACNA1C* and *COL1A1* genes previously suggested in the study from Camarero-Espinoza and Moroni^49^.

The different ES protocols employed resulted in distinct cellular responses, particularly regarding MSC gene expression profiles (Fig. 7). Considering the protocols STIM1 OM (1 h, Higher EF+Lower EF) and STIM2 OM (1 s, Higher EF), it appears that a single pulse of high EF (STIM2 OM) is sufficient to achieve *ALP*, *Runx2*, *COL1A1*, *CACNA1C* and *SCN1*$\alpha$ expressions similar to STIM1 OM. However, a more prolonged stimulation (prevalence of the Lower EF signal component) seems to be advantageous for higher expressions of more mature marker genes (*OPN* and *OC*) and mineralization (intense and spread Alizarin Red staining, Fig. 6), suggesting a more advanced differentiation stage achieved by the cells exposed to STIM1 OM than STIM2 OM. This effect is also observed comparing a single pulse of high EF (STIM2 OM) with multiple pulse protocols (STIM4 OM and STIM5 OM), in which the latter resulted in higher levels of bone-specific gene expression. Statistical significant differences in gene expression levels (*Runx2*, *OPN*, *OC* and *CANA1C*) were also observed between the protocols STIM4 OM (1 h, sequence of multiple Higher EF + short duration Lower EF) and STIM5 OM (1 h, sequence of multiple Higher EF + short duration Lower EF, with 5 times more pulses than STIM4 OM), suggesting the relevance of the signal period/frequency in the modulation of MSC osteogenic differentiation. Concordantly, Wang et al.^51^ study showed that different frequencies of ES lead to distinct outcomes in the in vitro osteogenesis of MC3T3-E1 pre-osteoblastic cells.

In general, our results (higher mineralization and *OPN* gene expression) suggest the benefits of using a current-controlled ES protocol (STIM OM3) for the in vitro stimulation of MSCs towards the bone lineage. We are aware that this improved performance in STIM3 OM may result from the potential signal oscillation at the electrodes that, when trying to support the prescribed current, increased the potential above 1.2 V, which probably generated uncontrolled and unknown redox artifacts that might influence MSC response. Previous studies have reported the role of reactive oxygen products on the enhancement of MSC osteogenic differentiation^22,52^. Despite this limitation, our work provides valuable insights towards the *in silico* and in vitro optimization of ES protocols for producing high-quality clinically relevant MSC-based BTE products for bone repair treatments.

Future studies should include a more profound characterization of the electrochemistry interface between the electrode and electrolyte, considering chronoamperometry and injected charge. Indirect probing methods should be privileged in the future to avoid direct influence in effects, like the Rogowski-coil method of measuring electric current^38^. Future work will include further optimization of the applied current-controlled ES protocols toward improved osteogenesis combined with whole transcriptome analysis. This will allow the unraveling and understanding of underlying molecular mechanisms/signaling pathways by which current-controlled ES modulates MSC osteogenesis. Developing new scalable devices for ES to allow a middle/high-throughput analysis should also be considered. Moreover, the future integration of direct-coupled ES systems within bioreactor systems will allow the improvement of the osteogenic differentiation process through a closer mimicry of the in vivo phenotype environmental conditions. Despite the large number of studies developing bioreactor systems for BTE strategies^53–56^, very few have explored the integration of ES as a strategy to improve the osteogenic differentiation of MSCs. To address this issue, our group has recently designed and fabricated an open-source bioreactor able to provide, simultaneously and in a controlled manner, capacitive-coupled ES and fluid-induced shear stress stimuli^57^. Preliminary results showed that the developed bioreactor supported the viability of MG-63 osteoblast-like cells cultured in 3D porous polycaprolactone scaffolds. However, future experiments are required to optimize the culture stimulation protocols (electrical and mechanical) and prove the bioreactor system’s ability to support long-term cultures and enhance the osteogenic differentiation of MSCs.

## Conclusion

In this work, a numerical FEM digital model of the culture platform was employed to characterize the system and predict the magnitude/distribution of the electric fields generated by the different DCoupled ES protocols. The models successfully assessed the impact of small culture medium volume and electrode geometrical variations on the delivered electric field. In vitro cell culture studies showed that all the ES protocols did not cause any impairment in cell viability and morphology and supported the osteogenic differentiation of hBM-MSCs. Importantly, our results suggest relevant differences when considering the applied protocol operation mode (potential versus current controlled), including the choice of stimulus duration and period. Our results highlight the performance of the applied current-controlled protocol (STIM3 OM) in promoting hBM-MSCs osteogenic differentiation as shown by a more efficient in vitro mineralization (higher calcium content and more intense Alizarin Red/Xylenol Orange stainings) and higher gene expression of the late osteogenic marker OPN. Overall, our study highlights the advantages of using computer modeling methods and robust electrical characterizations of the DCoupled setup in selecting and optimizing ES protocols to improve in vitro stem cell-based osteogenesis toward the development of novel therapeutic strategies for bone regeneration.

## Methods

### Direct coupled electrical stimulation system

**Figure 9:**
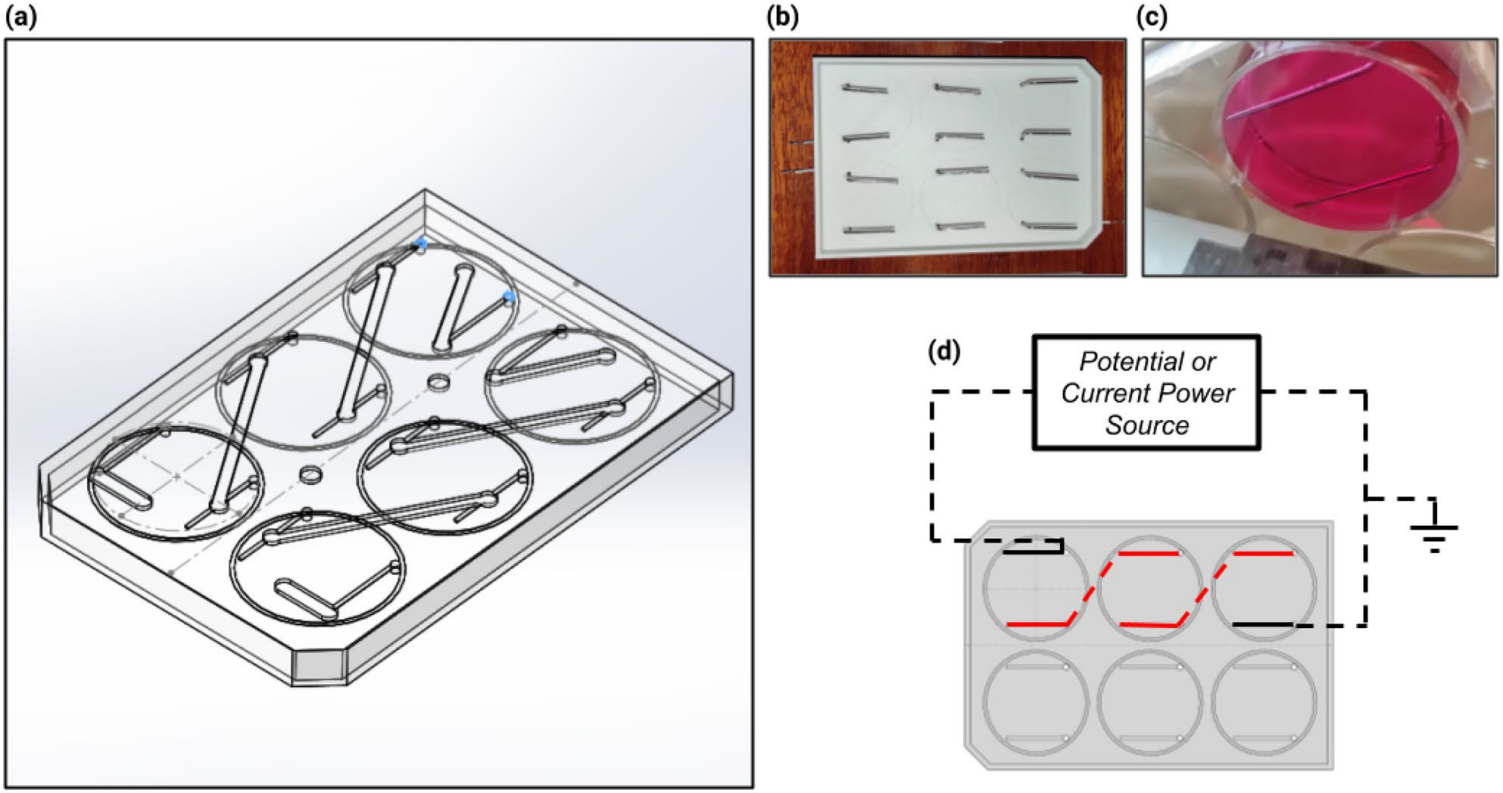
Developed Dcoupled electrical stimulation setup. (**a**) CAD of the developed custom lid; (**b**) Bottom view of the mounted medical grade stainless steel wire 316LVM electrodes; (**c**) Bottom view of a single well with electrodes, filled with culture media; (**d**) Electric connection schematic of the three wells connected in series.

Electrical stimulation system custom lids (see Fig. 9) for a 6-well plate polystyrene tissue culture-treated (Falcon®, Corning, USA) were designed and 3D printed in C8 material (3d4makers, Netherlands)^58^ by fused deposition modeling technique (Creatbot F430, Henan Suwei Electronic Technology Co., China). The custom lid computer-aided design files are available for download at Figshare (https://doi.org/10.6084/m9.figshare.23629926.v1). Medical grade stainless steel wire 316LVM (Tegra Medical, Franklin, USA), with a 1 mm diameter, was manually bent into an L-shape (width 22 mm, height 18 mm) and used as electrodes, similarly to Mobini et al.^28^. Each L-shaped electrode was inserted at its respective hole at the top of the custom lid and glued by a drop of commercial silicone, remaining separated by a distance of 25 mm to the next electrode in the same well. The described electrode pair per well constitutes the direct-coupled (DCoupled) ES system.

**Figure 10:**
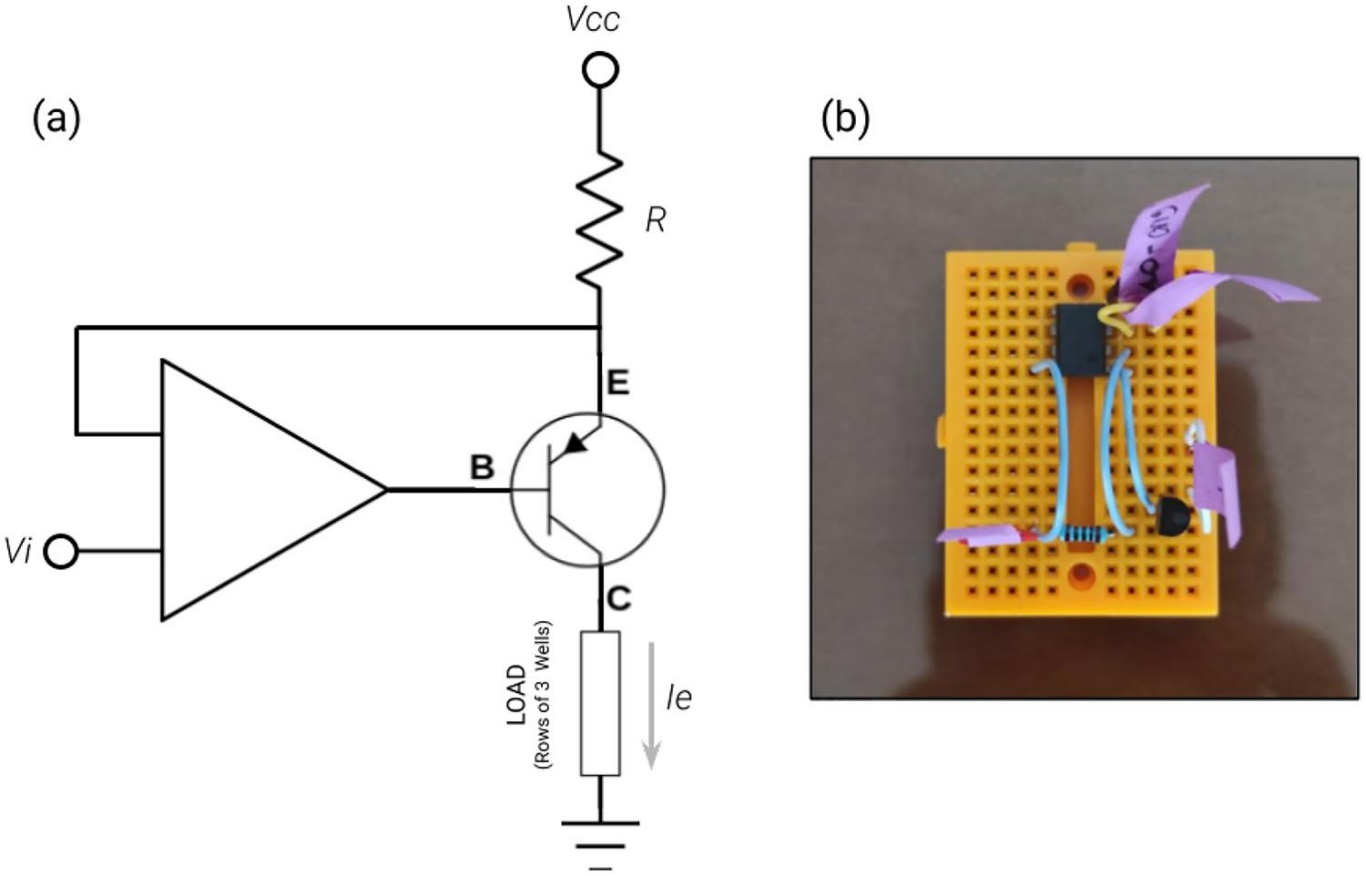
Custom current source electric circuit developed for the Dcoupled setup. (**a**) Electronic circuit schematic; (**b**) Image of the corresponding assembled breadboard with the required electronic components.

Every row of three wells was connected to guarantee that the same electrical current and, correspondingly, the same electric field were applied across these three wells. Figure 9d shows the electrical connection schematic. Each row (n=3) was then subjected to an electric potential or electric current, with duration and characteristics according to the required stimulation protocol to be applied. The electric potential step application, constant or intermittent, was performed by a lab power source equipment (Tektronix, Berkshire, UK). The electric current application was performed using a custom current source electric circuit (Fig. 10) composed of an operational amplifier (model TLV 2302), a PNP transistor (model PNP BC 556), and a 100-ohm resistor, wired accordingly with Fig. 10 schematic. This current pump electric circuit requires a power source of 12 V, a ground reference, and a potential input ($V_i$) that allows controlling and adjusting the electric current output of the pump circuit, accordingly with the following expression: $I_e=(V_{cc}-V_i)/R$ (see Fig. 10). Prior to the experimental assays, the output of each potential or current source condition was measured and validated with a multimeter (ISO-TECH IDM 73, Ahaus, Germany).

### Determination of culture medium electrical conductivity

Standard basal media (BM) is composed of Dulbecco’s modified eagle medium (DMEM, Gibco Thermofisher Scientific, Waltham, MA, USA) supplemented with 10% fetal bovine serum (FBS, MSC qualified, Gibco, Thermofisher Scientific) and 1% Antibiotic-antimycotic (Anti-Anti, Gibco, Thermofisher Scientific). Osteogenic culture media (OM) is composed of DMEM + 10% FBS(MSC) + 1% Anti-Anti supplemented with 10 mM beta-glycerolphosphate (Sigma-Aldrich, St. Louis, MO-IL, USA), 10 nM dexamethasone (Sigma-Aldrich) and 50 µg mL^−^^1^ ascorbic acid (Sigma-Aldrich). Standard basal culture medium and osteogenic medium electrical conductivities were measured at room temperature (21–23 °C) and 37 °C using a multimeter (ISO-TECH IDM 73).

### Direct coupled electrical stimulation system response characterization

The response of a single well from the developed direct-coupled system was studied to determine if the electrode/electrolyte interaction between the stainless steel wire 316LVM and the osteogenic culture medium or basal medium were driven by a faradaic or non-faradaic process. Following the procedure described in the work of Biesheuvel et al.^33^, we applied an electric current step input to one of the electrodes and fixed the other as ground. The electric potential at the first electrode was probed with an oscilloscope (Keysight DSOX 1102A, Santa Rosa, USA), checking if the resulting curve is likewise the faradaic or non-faradaic typical waveform, as explained by Biesheuvel and colleagues^33^. An electric potential step was also applied in the same single well, and the resulting potential waveform at the interface electrode/electrolyte was registered with the same oscilloscope.

Taking into consideration the results from the single well electric response characterization and to avoid water electrolysis, the response of a row of three wells in series to a potential step of 1.2 V was also acquired to check if the interpretation of a single well result could be expanded to three wells in a row configuration. A multimeter (KLEIN TOOLS MM600, Chicago, USA) was used to register the electric current passing through these three wells in series to the ground electrode (last well of the row). With this test setup, by applying a range of input potentials (from 0 to 4 V, at increasing steps of 0.5 V), we obtained an I-V curve for the system electrode/electrolyte interactions.

### Electric field predictions from finite element analysis

**Figure 11:**
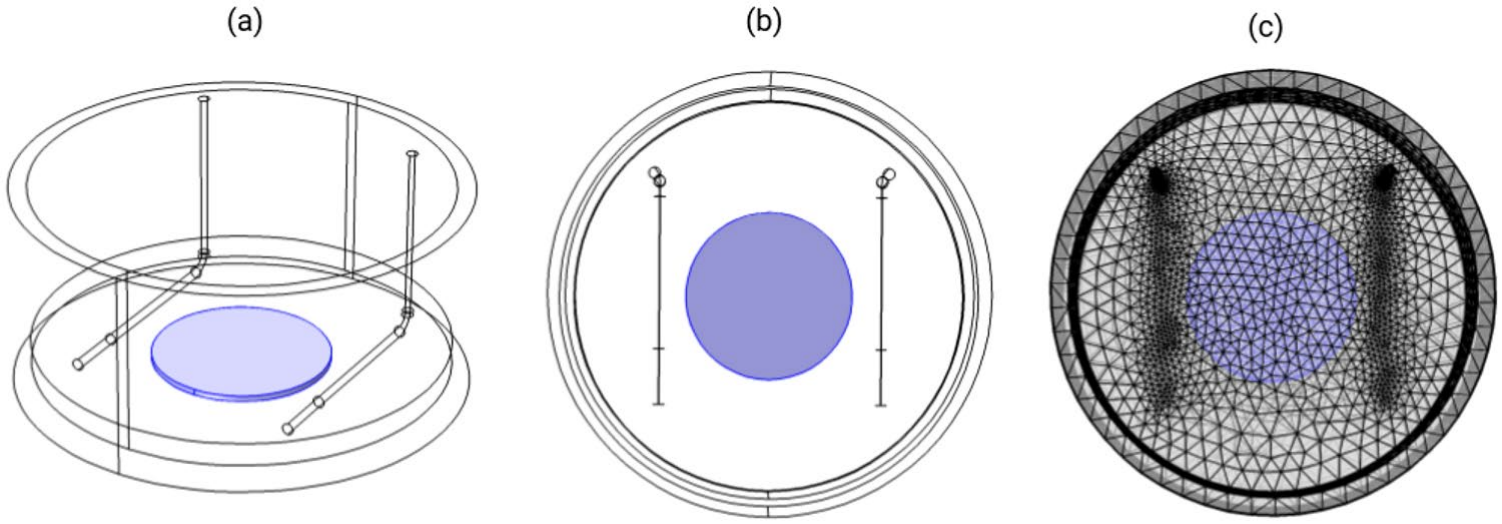
FEM model geometry of the developed DCoupled setup. The central disk, in blue, represents the region-of-interest (ROI) under study. (**a**) Perspective view of the geometry; (**b**) Top view of the geometry; (**c**) Top view of the fine mesh.

To predict the electric field delivered by the DCoupled system to the cell cultures, a finite element analysis was conducted with the AC/DC module of COMSOL Multiphysics (version 5.2a, www.comsol.com, Stockholm, Sweden). Considering a stationary study, the Electric Current (ec) physics interface was selected. A 3D physics-controlled mesh was generated in COMSOL with the finer mesh option. The model is composed of three material domains, characterized by their electrical conductivity ($\sigma$) and relative permittivity ($\epsilon _r$), according to Fig. 11: stainless steel 316LVM for electrodes ($\sigma$: $1\times 10^{5}$ S m^-1^, $\epsilon _r$: 1); osteogenic culture medium at 37 °C ($\sigma$: 1.725 S m^-1^ - determined experimentally as described above, $\epsilon _r$: 80.1^59^); polystyrene petri dish ($\sigma$: $6.7\times 10^{-14}$ S m^-1^, $\epsilon _r$: 2.5). An electric potential step of 1.2 V was applied to obtain the current values for the I–V curve (avoiding water electrolysis by staying below the 1.23 V limit^23^). Using this curve, we obtained a peak electric current of 0.17 mA, followed by a drop in current to 0.03 mA (see results section), both measures obtained for the osteogenic medium at 37 °C. These electric current conditions were then added to the model as a floating potential boundary condition to one of the electrodes while setting the other to a ground boundary condition. The average electric field was calculated at a disk shape region of interest, placed at the center of the well and equidistant from both electrodes (Fig. 11a, b, c). The solution of the model was computed using the conjugate gradients iterative solver.

Due to slight differences between electrodes’ sizes, angles, and distances generated when hand-mounted into the custom lid, multiple electrode geometries were modeled inside a single culture well to study and understand the impact of minor geometrical variations on the delivered electric field prediction. Three geometry variation studies were performed: (1) varying distance between electrodes from 20 to 25 mm while keeping the same culture medium height and a constant electric current; (2) varying culture medium heights, from 2 to 6 mm, while also comparing between regular or shorter electrodes lengths, $\delta L \pm 5$ mm (for this study, we kept the electric current constant at 0.05 mA); (3) comparison of the EF delivered between the regular position and the worst-case tilt scenario, where one of the electrodes has an upper tilt of 5°, and the other electrode has a bottom tilt of the same amplitude. This comparison was performed while keeping the same culture medium height of 5 mm and a constant electric current.

### Human MSCs culture

Human bone marrow-derived MSCs (hBM-MSCs) used in this work were part of the cell bank available at the Stem Cell Engineering Research Group - Institute for Bioengineering and Biosciences (iBB) at Instituto Superior Técnico (IST). hBM-MSCs were isolated according to protocols previously established at iBB-IST^60^. Bone marrow aspirates (Male 46 years) were obtained from Centro Clínico da GNR-Lisboa under previously established collaboration agreements with iBB-IST. An additional sample of fresh, unprocessed bone marrow (Male 24 years) was obtained from Lonza (Switzerland). The human samples were collected from healthy donors after written informed consent according to Directive 2004/23/EC of the European Parliament and of the Council of March 31, 2004, on setting standards of quality and safety for the donation, procurement, testing, processing, preservation, storage, and distribution of human tissues and cells (Portuguese Law 22/2007, June 29), with the approval of the Ethics Committee of the respective clinical institution. Isolated cells were frozen in liquid/vapor nitrogen tanks until further use. Before the cell culture assays, the hBM-MSCs were thawed and expanded on tissue culture flasks (T-75 cm^2^) using low-glucose DMEM (Gibco-Thermo Fisher Scientific, Waltham, USA) supplemented with 10% FBS (Gibco-Thermo Fisher Scientific, Waltham, USA) and 1% Anti-Anti Solution (Gibco-Thermo Fisher Scientific, Waltham, USA). hBM-MSCs were kept in an incubator at 37 °C and 5% CO_2_ in a humidified atmosphere, and the medium was exchanged every 3 days. All the experiments were conducted using cells between passages 3 and 5.

### Electrical stimulation protocol hypothesis

With the information from the electric field numerical predictions and with the DCoupled system electric response characterization, we created a group of electric stimulation conditions to test different hypotheses. Below, we describe the different electric stimulation protocols and their respective label:

- **CONTROL BM** = Without DCoupled stimulation, basal culture medium;
- **CONTROL OM** = Without DCoupled stimulation, osteogenic culture medium;
- **STIM1 OM** = 1.2V DC, constant, 1 h every 2 days, to test what is the biological impact of applying the DCoupled signal peak plus the signal dwell (Higher EF + Lower EF);
- **STIM2 OM** = 1.2V DC, constant, 1 s every 2 days, to test what is the biological impact of applying just the DCoupled signal peak (Higher EF);
- **STIM3 OM** = 0.03 mA DC, constant, 1 h every 2 days, to test what is the biological impact of applying a constant current condition (correspondent to the measured current of the signal dwell phase, this means we are just applying the Lower EF);
- **STIM4 OM** = 1.2V DC, ON/OFF with a period of 10 s, 1 h every 2 days, to test the biological impact of applying a condition similar to STIM2 OM, but with multiple DCoupled signal peaks and short time dwell phases;
- **STIM5 OM** = 1.2V DC, ON/OFF with a period of 2 s, 1 h every 2 days, to test the biological impact of applying a condition similar to STIM4 OM, but with five times more DCoupled signal peak phases and short time dwell phases.

Stimulation protocols were applied every 2 days from day 0 to day 14.

### Evaluation of the effects of different ES protocols on MSC proliferation and osteogenic differentiation

hBM-MSCs were harvested and seeded in 6-well culture plates at a density of 10.000 cells/ cm^2^. The cells were then cultured for 14 days in an incubator at 37 °C and 5% CO_2_ under the different electrical stimulation protocols and respective controls, as previously specified in the subsection “Electrical stimulation protocols hypothesis.” Culture media (volume of 3 mL per well) were fully renewed every 3 days. Cell morphology and metabolic activity were monitored throughout the culture. After 14 days of culture and exposure to the different ES protocols, the osteogenic differentiation of hBM-MSCs was assessed.

### Cell viability and morphology assessment

After 14 days of hBM-MSCs osteogenic differentiation under the different ES protocols, cell viability was assessed through LIVE/DEAD staining. Briefly, cells were first washed with PBS, after which they were incubated in the dark with ethidium bromide (2 µM) (Sigma-Aldrich) and calcein (4 µM) (Sigma-Aldrich) solution (prepared in PBS) for 1 h. Fluorescence images were obtained using a LEICA DMI3000B inverted fluorescence microscope (Leica Microsystems).

Cell morphology was observed and imaged at several culture time points using bright-field microscopy (LEICA DMI3000B, Leica Microsystems). At the end of the protocol (day 14), the morphology of the cells was also observed after staining with 4,6-diamidino-2-phenylindole dihydrochloride (DAPI, nuclei stains in blue) and Phalloidin (actin cytoskeleton stains in red). For that, the cultures were firstly washed with PBS, fixed in 4% paraformaldehyde (PFA; Sigma-Aldrich) solution (in PBS) for 20 min and permeabilized in a 0.1% Triton X-100 solution (Sigma-Aldrich) in PBS for 10 min. Afterwards, cells were incubated with Phalloidin-TRITC (2 µ g mL^1^ in PBS; Sigma Aldrich) for 45 min protected from light. Samples were washed with PBS and counterstained with DAPI (1.5 µ g mL^1^ in PBS; Sigma-Aldrich) for 5 min. Finally, the cultures were rewashed with PBS, and the fluorescence staining was imaged using an inverted fluorescence microscope (LEICA DMI3000B, Leica Microsystems).

### Metabolic activity assay

The metabolic activity of differentiating hBM-MSCs under the different ES protocols (and respective controls) was evaluated on days 3, 7, and 14 using the AlamarBlue assay (AlamarBlue Cell Viability Reagent; Thermo Fisher Scientific) following the manufacturer’s guidelines. Briefly, a 10% (v/v) AlamarBlue solution diluted in cell culture media was added to the cell cultures and incubated at 37 °C and 5% $\normalsize CO_{2}$ in a humidified atmosphere for 3 h. Fluorescence intensity was measured in a microplate reader (Infinite 200 Pro; TECAN) at an excitation/emission wavelength of 560/590 nm. For each experimental group, the fluorescence intensity was analyzed in 3 independent samples ($n=3$), and the fluorescence values of each sample were measured in triplicates.

### Alkaline phosphatase activity assay

Alkaline phosphatase (ALP) activity, associated with bone formation and osteoblast function, was quantified using a colorimetric ALP quantification kit (BioAssays Systems) following the manufacturer’s guidelines. ALP activity was assessed after 14 days of osteogenic differentiation under the different ES protocols. Cultures were firstly washed with PBS, and lysates were obtained after incubation in a 0.2% Triton X-100 (Sigma-Aldrich) solution (prepared in PBS) overnight at room temperature and under agitation. Afterward, a p-nitrophenyl solution (10 mM) was added to the lysates. The absorbance was measured on a microplate reader (Infinite 200 Pro; TECAN) at 405 nm. For each experimental group, the absorbance was quantified for three independent samples ($n=3$), and values for each sample were measured in triplicates. ALP activity values were calculated following the manufacturer’s protocol and normalized to the cell metabolic activity of each sample.

### Calcium quantification assay

The calcium content levels produced by hBM-MSCs under osteogenic differentiation and submitted to the different ES protocols for 14 days were determined using a calcium colorimetric assay kit (Sigma-Aldrich). First, cells were washed with PBS and incubated in a 1M HCl solution overnight (with agitation). Afterwards, the supernatant was collected and used for calcium content determination, following the manufacturer’s instructions. Briefly, three independent samples of each experimental condition ($n=3$) and diluted forms of a Calcium Standard Solution (500 mM) available in the kit were pipetted into a 96-well plate at several concentrations. Afterward, a Chromogenic Reagent and a Calcium Assay buffer (provided in the kit) were added to each well, and the solutions were gently mixed. The samples were incubated for 10 min in the dark at room temperature. The absorbance was measured on a microplate reader (Infinite 200 Pro; TECAN) at 575 nm (duplicate measurements per sample). The absorbance measurements for the different Calcium Standard Solution concentrations were used to develop a calibration curve, which was used to estimate the concentration of calcium present in each sample. The values were normalized to the cell metabolic activity of the respective sample.

### Osteogenic stainings

ALP/Von Kossa, Alizarin Red, and Xylenol Orange stainings are often used to confirm the osteogenic differentiation of hBM-MSCs by detecting the bone ECM markers (ALP and mineral deposits). In this study, the stainings were performed after 14 days of osteogenic differentiation under the different ES protocols. After being washed with PBS and fixed with 4% PFA for 20 min, the hBM-MSCs were first washed twice with milliQ and stained for ALP presence by incubation in a solution comprised of 0.1 M TRIS-HCl (Sigma-Aldrich), containing Fast Violet Solution (Sigma-Aldrich) and Naphthol AS MX-P04 (Sigma-Aldrich) for 45 min. The cells were washed three times with PBS and kept in milliQ water while observed under the microscope (LEICA DMI3000B, Leica Microsystems). After washing the samples with PBS, Von Kossa staining was performed on the same samples by incubating them in a 2.5% silver nitrate solution (Sigma-Aldrich) for 30 min. Finally, the cultures were washed three times with PBS, once with miliQ water, and kept in milliQ water until observation under the microscope (LEICA DMI3000B).

Alizarin red staining of the cells from different experimental groups was performed to detect the calcium deposits. PFA-fixed samples were incubated in a 2% Alizarin red (Sigma-Aldrich) solution (in PBS) for 1 h at room temperature. Afterwards, the cultures were washed multiple times with PBS and milliQ water, after which they were imaged with an inverted microscope (LEICA DMI3000B).

To further confirm the presence of mineral deposits within the samples after 14 days of hBM-MSCs osteogenic differentiation under the different ES protocols, a 20 mM Xylenol Orange solution (Sigma-Aldrich) was added to previously fixed samples for 1 h at room temperature. Cells were then washed successively with PBS, and the fluorescence staining was imaged using an inverted fluorescence microscope (LEICA DMI3000B).

### RNA isolation, conversion to cDNA, and quantitative real-time polymerase chain reaction (RT-qPCR) analysis

After the osteogenic differentiation of hBM-MSCs under the different ESTIM protocols for 14 days, cells were harvested from the plates, centrifuged, and the obtained pellets were stored at −  80 °C until further use. RNA extraction from cell pellets was performed using the RNeasy Mini Kit (QIAGEN) according to the manufacturer’s guidelines. Afterward, the RNA concentration of the different samples was quantified using a NanoVue Plus spectrophotometer (GE Healthcare).

According to the manufacturer’s protocol, cDNA was synthesized from the purified RNA using the High-Capacity cDNA Reverse Transcription Kit (Applied Biosystems). Reaction mixtures comprised of 10 µL of MasterMix–constituted by 2  µL of RT 10x buffer, 0.8  µL of dNTP mix, 4.2  µL of RNase-free water, 2  µL of random primers and 1  µL of Multiscribe Reverse Transcriptase—and 10  µL of purified RNA sample were mixed and incubated in a T100TM thermal cycler (Bio-Rad) for 10 min at 25 °C, 120 min at 37 °C and 5 min at 85 °C, and then were maintained at 4 °C.

RT-qPCR analysis was performed using a StepOnePlus real-time PCR system (Applied Biosystems) and NZYSpeedy qPCR Green Master Mix (2x), ROX plus (Nzytech) following the manufacturer’s protocol. The reactions were carried out at 95 °C for 10 min, followed by 40 cycles of 95 °C for 15 s and 60 °C for 1 min. All samples were analyzed in triplicates ($n=3$). Gene expression analysis was performed following the MIQE guidelines^61^. The results obtained were analyzed using the $2^{-{\Delta \Delta }Ct}$ method to determine relative changes in specific target genes (*ALP*, *Runx2*, *COL1A1*, *OPN*, *OC*, *CACNA1C* and *SCN1*${\alpha }$) expression compared with the control sample (hBM-MSCs at day 0 before seeding). Gene expression was primarily normalized against two reference genes (glyceraldehyde-3-phosphate (GADPH) and ribosomal protein L13A (RPL13A), and then calculated as a fold-change relative to the baseline expression of the target genes in the control sample. The primer sequences used in the RT-qPCR analysis are presented in Table 1.

**Table 1:** Primer sequences used in the RT-qPCR analysis.

GENE	FWD PRIMER SEQUENCE	REV PRIMER SEQUENCE
*GAPDH*	5′-GGTCACCAGGGCTGCTTTTA-3′	5′-CCTGGAAGATGGTGATGGGA -3′
*RPL13A1*	5′-GTGCGAGGTATGCTGCC-3′	5′-GCTTCAGACGCACGACCTT-3′
*ALP*	5′- ACCATTCCCACGTCTTCACATTT-3′	5′- AGACATTCTCTCGTTCACCGCC-3′
*Runx2*	5′-AGATGATGACACTGCCACCTCTG-3′	5′-GGGATGAAATGCTTGGGAACT-3′
*COL1A1*	5′-CATCTCCCCTTCGTTTTTGA-3′	5′-CCAAATCCGATGTTTCTGCT-3′
*OPN*	5′-CAGGTCTGCGAAACTTCTTAG-3′	5′-CTCCATTGACTCGAACGACTC-3′
*OC*	5′-TGTGAGCTCAATCCGGCATGT-3′	5′-CCGATAGGCCTCCTGAAGC-3′
*CACNA1C*	5′-GTACAAAGACGGGGAGGTTGAC-3′	5′-GTAGTTGTAGATGGGGCCCTTG-3′
*SCN1*${\alpha }$	5′- TTGTGACGCTTAGCCTGGTAG-3′	5′- ACGATGATGGCCAAGACGAG-3′

### Immunofluorescence analysis of bone-specific proteins

The presence of type I collagen (COL I), osteopontin (OPN), and osteocalcin (OC) (relevant proteins produced during bone ECM formation) within the cultures after 14 days of osteogenic differentiation under the different ES protocols was evaluated by immunofluorescence analysis. Previously fixed (PFA 4% for 20 min) samples were washed twice in PBS, after which they were immersed in a permeabilization/blocking solution comprised of 1% BSA (Sigma-Aldrich), 10% FBS and 0.03% Triton X-100 for 45 min at room temperature. Solutions containing primary antibodies for type I collagen (MA1-26771, Thermo-Fischer), osteopontin (ab8448, Abcam) and osteocalcin (MAB1419, R &D Systems) (1:200 in 1% BSA, 10% FBS, 0.03% Triton X-100 solution) were then incubated with the respective samples overnight at 4 °C. Cells were then incubated with the secondary antibodies (1:200 in 1% BSA; goat anti-mouse IgG-AlexaFluor 546 (Thermo Fisher Scientific) for COL I and goat anti-rabbit IgG-AlexaFluor 546 (Thermo Fisher Scientific) for OPN and OC) for 1 h at room temperature in the dark. Following two washes with PBS, the samples were counterstained with DAPI (1.5 µ g mL^1^ in PBS) for 5 min at room temperature, washed twice with PBS and imaged using a fluorescence microscope (LEICA DMI3000B).

### Statistical analysis

When applicable, results are presented as average values ± standard deviation (SD). All the in vitro cell culture assays were performed using three independent samples ($n = 3$) from two different donors (two independent experiments) unless specified otherwise. Statistical analysis of the data was performed by the Krustal-Wallis test followed by a Dunn’s post hoc test for multiple comparisons using the GraphPad Prism 7.0 software (GraphPad, San Diego, CA, USA). Data were considered statistically significant when the p-values obtained were less than 0.05 (95% confidence intervals, $*p < 0.05$).

### Supplementary Information

Supplementary Information.

## Data Availability

The datasets generated during and/or analyzed during the current study are available from the corresponding author upon reasonable request.
