# Supplementary material for: Direct coupled electrical stimulation towards improved osteogenic differentiation of human mesenchymal stem/stromal cells: a comparative study of different protocols
Source: Sci Rep. 2024 Mar 5;14:5458. doi: 10.1038/s41598-024-55234-y (PMC10915174; doi:10.1038/s41598-024-55234-y)
Supplement: Supplementary file 1 — Supplementary Information. [file 41598_2024_55234_MOESM1_ESM.pdf]

Direct coupled electrical stimulation towards improved osteogenic differentiation of human mesenchymal stem/stromal cells: a comparative study of different protocols.

MOBINI *ET AL.* (2016) DCOUPLED SETUP

Mobini et al. 2016, Considerations on geometrical model design.

Mobini *et al.* (2016) utilized a 6-well cell culture plate (128x85x22 mm) produced by TPP (Transadingen, Switzerland). Unfortunately, this well-plate model is discontinued for the reported dimensions with a dish diameter of 33.78 mm. All the remaining models from the same supplier are produced in polystyrene (PS). The electrode wire was reported with a diameter of 1 mm and was made from 99.99% of pure platinum. This wire with a length of 50 mm was bent into an L-shape configuration with 29-21 mm parts. Two electrodes per culture dish were separated by 25 mm. We estimate a wall thickness of 1 mm for the entire dish based on recent petri dish models. We assumed the reported maximum plate height reported by the supplier, 22 mm (the real dimension should have been shorter). Also, regarding the reported distance between electrodes, we assumed from the wire center axis to the other wire center axis. The distance of the electrodes to the dish bottom is not reported in the original manuscript and was assumed to be 0.25 mm. The culture medium volume used in each culture dish well was not reported. Still, for similar culture dish dimensions, the Thermo Fisher table recommends a culture medium volume of 2 mL for this 6-well culture plate size, and this was the value we used in the geometrical model, obtaining the liquid volume height of 2.26 mm. Figure S1 resumes the geometrical model dimensions for the Mobini *et al.* (2016) setup.

[https://www.thermofisher.com/pt/en/home/references/gibco-cell-culture-basics/cell-culture-protocols/cell-culture-useful-numbers.html]

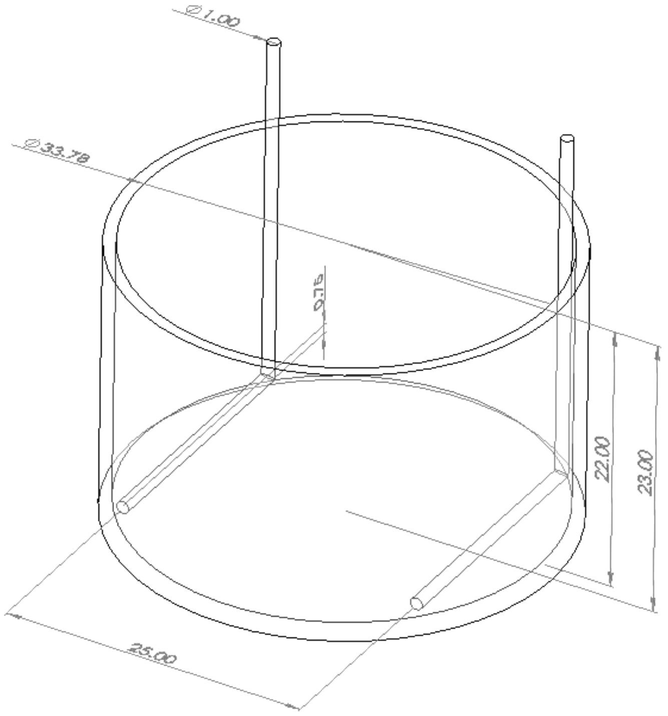

Figure S1 - The considered geometry of a single well according to the data reported by Mobini et al. (2016), including the described assumptions where information was lacking. Measurement values are in millimeters.

Mobini et al. 2016, Considerations on FEM modeling in COMSOL.

The geometric model described in the previous step was imported into COMSOL. Since the culture dish, electrode materials, and culture medium electrical properties were not measured, estimated, or reported in the Mobini et al. (2016) manuscript, approximate values for those properties were obtained from the literature. Electrical properties for the culture medium were obtained from *Visione et al. (2018),* considering an electric conductivity of 1.5 S/m and a relative permittivity of 80.1. Polystyrene material electrical properties were retrieved from *professionalplastics.com* with an electrical conductivity of 10^{-18} S/m (reported >10^{18} Ohm/m) and an electrical relative permittivity of 2.6 (interval 2.4 to 3.1). Platinum material electrical conductivity was considered with a value of 9.52×10^{6} S/m (reported 1.05×10^{-9} Ohm/m), and relative permittivity for all metals at lower frequencies is considered 1. The electric stimulus applied in Mobini *et al.* (2016) setup was a 2.2 V direct current, generating a reported electric field of 100 mV/mm for that input electrical potential. To translate this to COMSOL, two boundary conditions were applied, one for ground in one electrode tip and another considering the 0.07 mA current condition DC floating potential on the opposite electrode tip (as reported later by Srirussamee *et al.*

(2019) directly measuring it on Mobini's setup). A physics-controlled mesh with normal size was generated that consists of 19320 domain elements, 8080 boundary elements, and 721 edge elements. The solution converged in 2 seconds and calculated a volumetric average electric field in the cell culture medium of 0.59 V/m.

DEVELOPED DCOUPLED SETUP

CAD and COMSOL files availability.

All developed CAD geometries and COMSOL models are available in Figshare for download at the following link: *https://doi.org/10.6084/m9.figshare.23629926.v1*

Considerations on modeling geometrical variations in the developed setup.

The impact of varying electrode size, tilt position, and culture medium volume was evaluated with COMSOL for the developed one-well model. The results are summarized in the following tables A, B, and C. All values consist of the volumetric average estimates at the region of interest (a disk shape region with a radius of 7.5 mm and a height of 0.5 mm, placed in the center and bottom of the cell culture well). All studies were modeled in basal medium at 37°C.

Table A - Impact of different distances between electrodes and input current in the electric field, for a fixed culture medium height of 2 mm.

Distance Between Electrodes	Electric Current	
	0.03 mA	0.17 mA
25 mm	0.33 V/m	1.88 V/m
20 mm	0.33 V/m	1.88 V/m

Table B - Impact of different well liquid volumes in the electric field for a constant electric current of 0.05 mA. The green values indicate the variation in the EF due to liquid volume change, while the red values indicate the variation imposed by the longer versus shorter electrodes (+- 5 mm size).

Liquid Height	Electric Field, Long Electrodes	Electric Field, Short Electrodes
2 mm	0.55 V/m	0.58 V/m (+5%)
3 mm	0.37 V/m (-32%)	0.38 V/m (+3%)
4 mm	0.27 V/m (-27%)	0.28 V/m (+4%)
5 mm	0.22 V/m (-18%)	0.23 V/m (+5%)
6 mm	0.18 V/m (-18%)	0.19 V/m (+6%)

Table C - Worst case scenario for a twist angle 5° up/down for a fixed culture medium height of 5 mm. The percentage difference from the no-twist scenario under the same conditions is represented in red.

Electric Current	Electric Field
0.03 mA	0.133 V/m (0.09%)
0.17 mA	0.75 V/m (0.09%)

Oscilloscope measurements.

Here we made available in high resolution the print screens from the oscilloscope measurements that are part of the main manuscript Figure 4 and Figure 5.

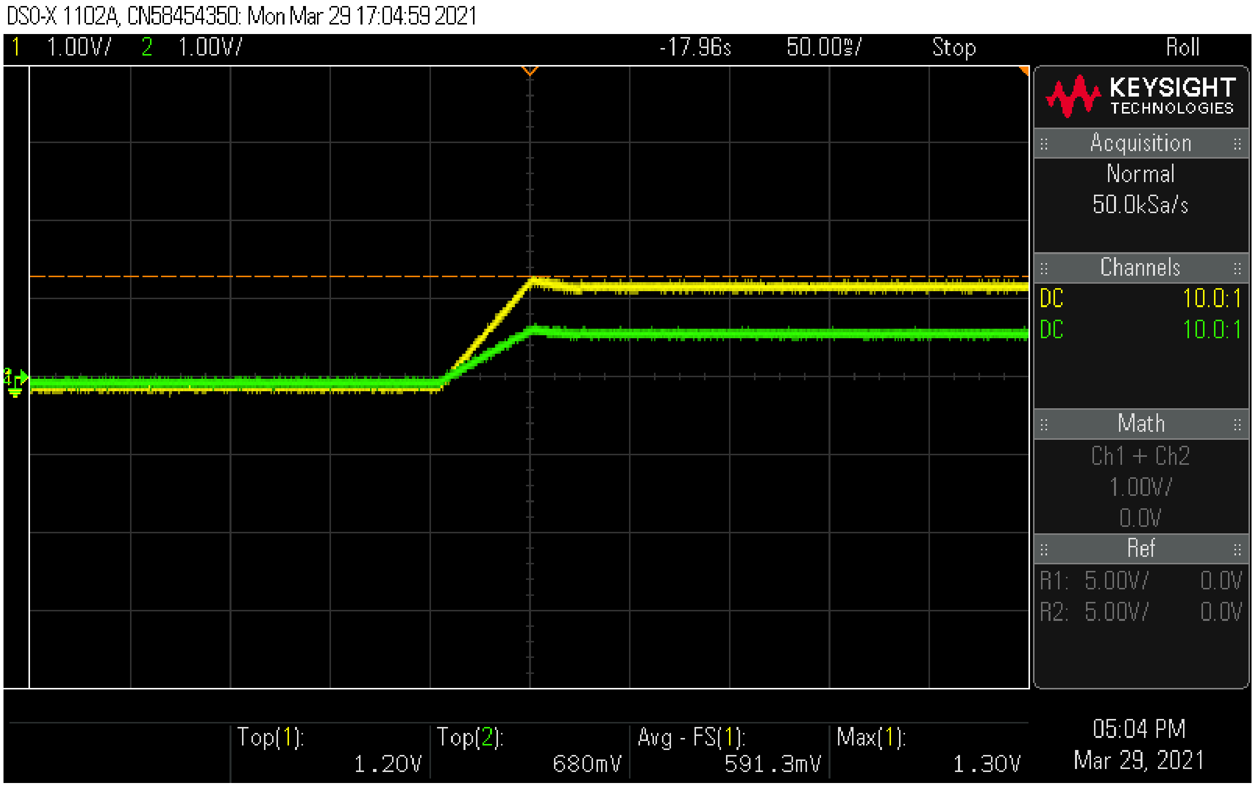

Figure S2 - Oscilloscope measurement for the electric potential drop between three wells in series. This is the response of the developed DCoupled system for the application of a potential step waveform.

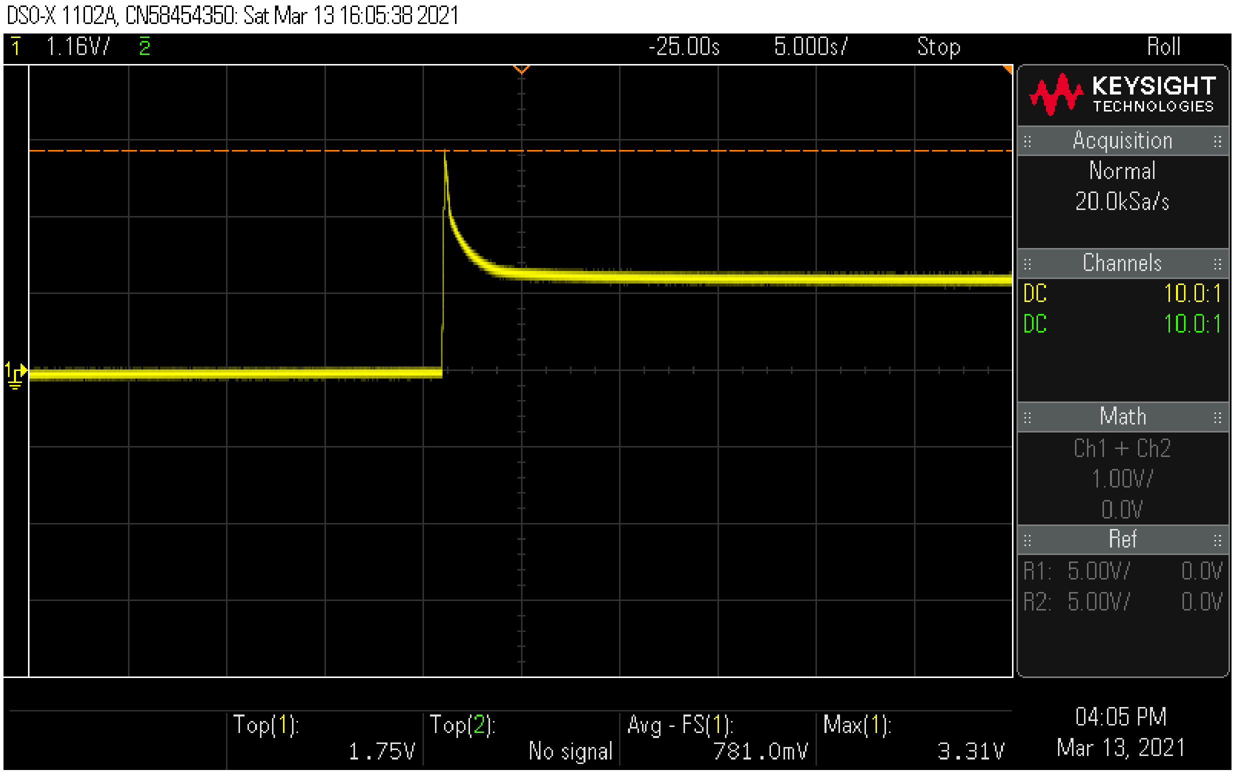

Figure S3 - Oscilloscope measurement for the electric potential drop for a single well. This is the response of the developed DCoupled system for the application of a potential step waveform.

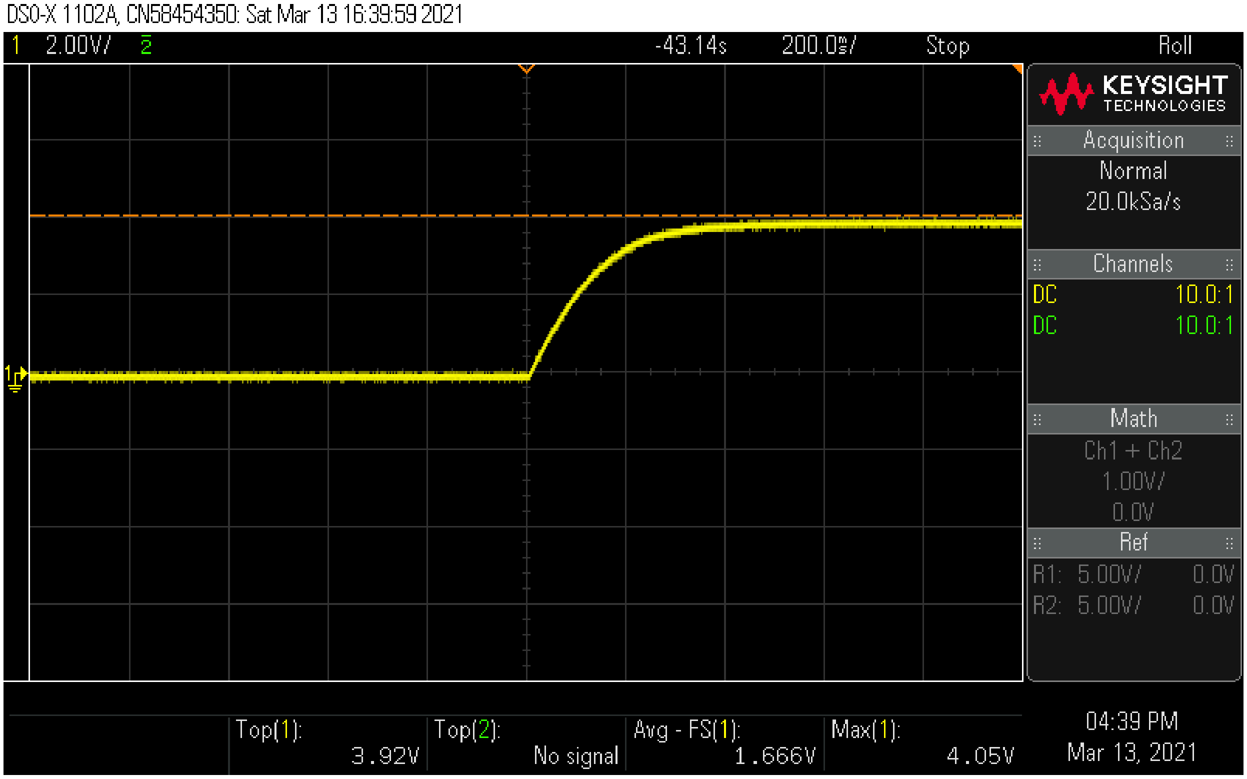

Figure S4 - Oscilloscope measurement for the electric potential drop for a single well. This is the response of the developed DCoupled system for the application of a current step waveform.

Stability of reference genes expression between the different experimental groups

The normalization of the RT-qPCR results using two reference genes (*GAPDH* and *RPL13A*) was done by determining the geometric mean of these genes and then normalizing the target genes expression to the calculated geometric mean (as reported in [REF S1])

[REF S1] *Vandesompele, J., De Preter, K., Pattyn, F., Poppe, B., Van Roy, N., De Paepe, A., Speleman, F. Accurate normalization of real-time quantitative RT-PCR data by geometric averaging of multiple internal control genes. Genome Biol. 3, 1-12 (2002), doi: 10.1186/gb-2002-3-7-research0034*

Besides, the normalization of the target genes expression against the two reference genes (*GAPDH* and *RPL13A*), additional RT-qPCR experiments were performed to test the expression stability under the different experimental conditions studies. As it is possible to observe in Table D, the CT values obtained for the two reference genes (*GADPH* and *RPL13A*) are quite similar for all the experimental conditions described (variations in CT values between the two reference genes are shallow (all < 3.1%)), which suggests that the reference genes are stably expressed between the different treatment groups, confirming the reliability of the RT-qPCR methods and results presented by the authors.

This suggests the effects of normalizing our target gene expression also against the *RPL13A* reference gene on our obtained results were minimal.

Table D - Average CT values (from n=3 independent samples) and respective percentage of variation of CT values calculated from the RT-qPCR data to compare the two different reference genes (*GADPH* and *RPL13A*) and confirm the stability of their expressions between the different treatment groups.

Experimental Conditions	Reference Gene	Average CT Values	% Variation CTValues (in relation to GAPDH)
hBMSC (Day 0)	*GADPH*	18.99 ± 0.07	1.05 %
	RPL13A	19.19 ± 0.02	
Control BM	*GADPH*	18.31 ± 0.06	2.35 %
	RPL13A	18.74 ± 0.01	
Control OM	*GADPH*	19.21 ± 0.01	3.07 %
	RPL13A	18.62 ± 0.04	
STIM1 OM	*GADPH*	19.17 ± 0.03	1.88 %
	RPL13A	19.53 ± 0.06	
STIM2 OM	*GADPH*	19.40 ± 0.01	1.24 %
	RPL13A	19.69 ± 0.04	
STIM3 OM	*GADPH*	19.66 ± 0.01	0.51 %
	RPL13A	19.76 ± 0.07	
STIM4 OM	*GADPH*	19.22 ± 0.01	0.10 %
	RPL13A	19.24 ± 0.01	
STIM5 OM	*GADPH*	19.08 ± 0.23	1.94 %
	RPL13A	19.45 ± 0.19	
